# The depth structure of a good birth: reconfiguring the environment in a high-risk labour ward birth and creating sanctuary behind a screen

**DOI:** 10.3389/fgwh.2025.1610077

**Published:** 2025-08-07

**Authors:** Jane Clossick

**Affiliations:** AAD Cities, School of Art, Architecture and Design, London Metropolitan University, London, United Kingdom

**Keywords:** high-risk birth, birth environments, physiological birth, birth unit design, birth territories, obstetric care, midwifery care, birthing people’s autonomy

## Abstract

This article explores how the spatial, relational, and sensory conditions within an obstetric-led hospital birth room were subtly reconfigured to support a safe, satisfying birth, even though the birth in question was considered high risk. Drawing on autoethnographic reflections and interviews with caregivers from the author's own birth at the National Health Service Royal London Hospital, the paper examines the transformation of a standard labour ward room through a low-tech intervention: the erection of a cloth screen brought from home. This simple act created a distinct spatial zone in which institutional norms were less prevalent, fostering privacy, autonomy, and integrative care practices that protected physiological labour and enhanced maternal agency. The article situates this personal narrative within broader theoretical frameworks of birth territory, sociospatial theory, environmental psychology, and institutional power, arguing that space and care interact in complex ways to shape birth experiences. It contributes to calls for more humanised, woman-centred approaches to birth architecture and practice, particularly in highly techno-rational and medicalised settings, and proposes that even small acts of spatial resistance have the potential to generate meaningful shifts in care culture.

## 1 Introduction

**Figure 1:**
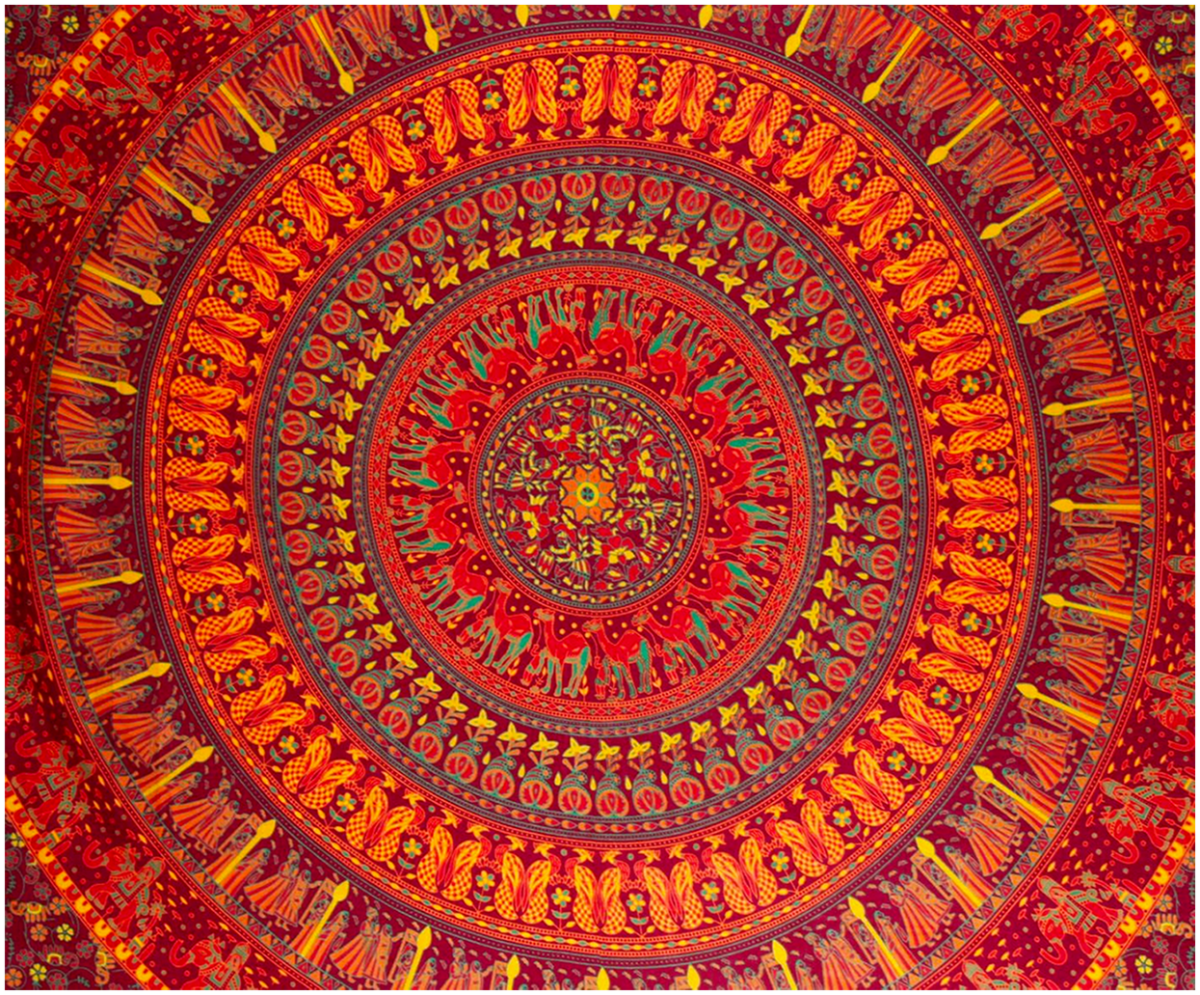
The pattern on the 2 m×2 m cloth which I used as a screen to divide the birth room at the Royal London Hospital, pegged onto two drip stands. Photograph by the author.

In 2021, I gave birth to premature twins in a high-risk labour room at the Royal London Hospital. Determined to exercise my agency, I brought with me a 2 m^2^ piece of printed cotton fabric, shown in Figure 1, with the intention of creating a private space, or a den, in the labour room. As an academic architect with an interest in the sociospatial structuring of human experience, I suspected that this intervention might increase the likelihood of my experiencing a safe, satisfying birth. This article spatially analyses what happened during the unmedicated vaginal birth of Twin 1, Julian, which was ‘outside guidance’. It is intended as a springboard for future research into the same topic and, based on a single case study, presents the hypothesis that spatial, relational, and sensory conditions within obstetric-led hospital birth rooms can be subtly reconfigured to better support safe, satisfying birth.

The text is structured as follows: Section 2 is an introduction to literature as a backdrop to this case study, about what is already known about the relationship between birth environments and safe, satisfying birth and why such conditions are often difficult to achieve for people categorised as ‘high risk’. Section 3 is the methodology of the case study and its underpinning sociospatial assumptions. Section 4 is a narrative birth story, based on the birth notes obtained from the hospital, along with my own recollections, those of my husband Colin, our doula Becky, and the obstetrician Philippa. Section 5 is a series of reflections on the three key spaces which feature in the birth story: the bed, the bathroom, and the screened birthspace and how these spaces interacted with caregiving practices and institutional norms at the Royal London in ways which resulted in my experiencing a safe, satisfying birth. Finally, in the conclusion (Section 6), some implications are suggested for future design and research.

## 2 Birth environments for a safe, satisfying birth

Ensuring a positive birth experience benefits not only the birthing person but also the baby, caregivers, and society. Safe, satisfying birth (SSB) is one in which no one is harmed physically or psychologically, and where the birthing person feels untraumatised by the experience. It is often correlated with physiological birth, characterised by spontaneous labour onset and minimal intervention (1), although they are not always the same. SSB is more likely if the birth experience includes physiological labour as hormones released during it such as oxytocin support infant/parent attachment (2, 3). SSB supports parental wellbeing and infant health because birth has lasting psychological effects; those who feel respected and safe report greater fulfilment and lower postpartum depression (4) while a traumatic childbirth experience can result in posttraumatic stress disorder (PTSD), fear of childbirth, and disrupted bonding (5). The benefits of SSB extend beyond individuals; woman-centred care is a human right (4) and key to reducing unnecessary interventions that burden healthcare systems.

Woman-centred care contributes to SSB. A birth environment is composed of physical space (objects, decor) as well as people, and it is situated in the wider structures of society and its institution(s), and the human and non-human components of birth environments interact with one another, shaping the experiences of all occupants. Foureur developed a conceptual model describing the relationships among the set of variables in a birth environment: safe, satisfying birth is a function of the ‘birthing person’s stress’ and ‘communication’ with the birthing person multiplied by ‘staff stress and communication’ mediated by ‘birth unit design’ and ‘model of care’ (6). Lefebvre (7) argues that spaces are always imbued with social and ideological meaning, and this includes birth environments. Nations, institutions, corporations, or individuals claim ownership of different types of birth environment, shaping their characteristics, accessibility, and meaning (8). Institutional norms of birth caregiving practices as well as acceptable behaviour of birthing people are communicated by the environment in multiple ways. Caregiver philosophy, continuous labour support, and communication dynamics significantly shape the culture of birth (9, 10). Humanised care, particularly in high-risk births, prioritises shared decision-making and emotional safety (11), reinforcing the idea that the emotional climate co-created by companions, staff, and the wider institution is just as critical to SSB as the physical environment.

### 2.1 The impact of the birth environment on birthing people and babies

The birth environment seems to play a crucial role in shaping birth outcomes and experiences, and whether SSB is achieved. Birth room design has been shown to influence physiological responses, including the production of oxytocin, a key hormone in labour and emotional bonding (5). A considerable proportion of birthing people express a preference for a cosy and familiar birthing environment (12–15). This ‘homely’ birth setting may be described as the opposite of a hospital environment. Architecture and design can facilitate behaviours known to enhance wellbeing during labour, such as free movement (12), adopting varied positions (9, 16), personalising the space (17), capacity for relaxation (18, 19), feeling comfortable (20), and engendering a sense of privacy and protection (21, 22). When birthing people are protected, can move freely, and can personalise their space, they are more likely to experience reduced stress and improved labour progression and more likely to achieve SSB.

Two systematic reviews on the effects of birthing room design on maternal and neonatal outcomes have been carried out, although both comment that available evidence is scant and a safe parent and baby does not necessarily mean that the birthing person experienced SSB. A systematic review by Nilsson et al. (5) found that optimal spatial conditions include ‘means of distraction, comfort, and relaxation’, temperature, ‘features of familiarity’ (things from home), and ‘diminishing a technocratic environment’. Sands et al. (23), in their review of birth environments for people with complex pregnancies, found that features such as adaptability, spaciousness, and comfort can support staff in assisting birthing people to adopt more comfortable positions, facilitating more straightforward births. Birthing people valued access to birthing pools and supportive tools such as floor mats or bean bags, as well as the freedom to move during labour. A key preference expressed was for a private, homely space where they could control access and feel shielded from the view of others.

However, spatial environments in which SSB can be more difficult to achieve include obstetric-led units where continuous foetal monitoring and oxytocin infusions may restrict mobility (23). The study by Sands et al. (23) also highlighted that birth environments for high-risk labour are shaped by competing priorities between birthing people, midwives, and obstetricians, which can create tension in how space is designed and used. The findings of these studies are echoed throughout the qualitative literature (15, 24–27). In terms of quantitative studies, for birthing people with low-risk pregnancies who birth in midwifery-led units, which tend to have the qualities listed, there are lower medical intervention rates without increased risk to mothers or babies (28). No large-scale cohort-level data exists, however, for the impact of birth environment on high-risk labour and birth.

### 2.2 The impact of the birth environment on caregivers

The behaviour of companions and staff plays a pivotal role in shaping safe, satisfying birth experiences. High-quality midwifery care, marked by continuous support and respect for physiological processes, is strongly associated with improved maternal and neonatal outcomes (29). The people who comprise the birth environment also reflect and reinforce a particular culture of care (10), and as labour intensifies, birthing people often become less aware of spatial design and increasingly reliant on caregivers (30). In birth environments that host ‘integrative power’ (22), where the birthing person is the key decision-maker, through midwifery guardianship and respect for bodily sensations, birthing people retain their agency. Conversely, environments steeped in ‘disintegrative power’, where the birthing person is coerced or forced by caregivers, promote passivity, especially when interventions are framed as essential or when time pressure dominates (24).

Most births take place in hospitals, institutions oriented towards treating illness. In such settings, physicians trained to manage complications are more likely to use interventions. Midwifery-led settings typically involve fewer interventions, as midwives are more likely to support physiological birth (10). The values and philosophies of care providers, the presence of doulas, and staff willingness to offer continuous support shape the culture of the birth environment. This culture directly impacts communication, safety, and outcomes for birthing people (9, 10). Supportive caregivers who offer privacy, reduce interruptions, and protect the ‘birth bubble’ allow birthing people to relax and let go of fear, even in clinical settings (31). In contrast, surveillance and authoritative control create anxiety and disempowerment and increase the likelihood of PTSD.

Although UK policy suggests everyone should have a choice in birthplace, people with high-risk pregnancies, estimated at 15%–20% of all pregnancies (32), are typically required to birth in obstetric-led units where safety concerns dominate care practices. In these settings, the definition of ‘optimal care’ is largely medicalised, prioritising continuous monitoring and rapid access to intervention (11, 33). Driven by clinical safety, it often compromises psychological and emotional wellbeing. People categorised as high-risk report heightened anxiety and emotional distress (34), exacerbated by feelings of vulnerability and disempowerment from the ‘high-risk’ label (33, 35).

Key features of humanised birth, such as privacy, autonomy, and environmental comfort, are often missing in obstetric units. Features such as natural light, control over space, and noise reduction are frequently absent, contributing to emotional discomfort and disrupted hormonal regulation essential for labour (23). Structural barriers, including legal liability concerns, diminished midwifery authority, and physician-led decision-making, may further inhibit personalised, respectful care (11). Although alternative birth environments have been shown to support normal birth (14), they remain largely inaccessible to high-risk populations. In addition, most births in the UK (86%) still occur in obstetric-led units (23, 36), due to preference, limited choice, or labour transfer (23), with even higher rates in the USA (98.4%) and Australia (93.6%) (37, 38). Although up to 62% of birthing people may require obstetric care due to complications (39), it is not clear whether fewer would require help if hospital birth environments were more conducive to promoting physiological birth. The sheer numbers of births in hospitals, combined with evidence that birth environment is linked to SSB, underscore the urgent need for humanised sociospatial approaches to design within these medicalised settings.

### 2.3 Gaps in the literature about birth environments and birth outcomes

Despite increasing awareness of how birth environments influence maternal and neonatal outcomes, several research gaps remain. First, there is a need for design-related research that goes beyond the exclusionary confines of quantitative, experimental studies typically associated with healthcare facility design (30, 40). Many studies fail to clearly distinguish between spatial structuring and spatial aesthetics, obscuring how specific environmental factors operate (22). Moreover, the mechanisms by which environmental benefits affect labour and birth remain largely untheorised, contributing to the perception that positive spatial features are luxuries rather than essential supports for physiological birth (22). Although tools such as the Birth Unit Spatial Evaluation Tool (BUDSET) have been developed (9), there is still no widely tested and adopted instrument for measuring the qualities of birth environments. Observational research, such as Joyce or Lepori's studies on home birth behaviours, has highlighted the value of returning to fundamental design principles grounded in spontaneous maternal behaviour (41, 42). Yet, interdisciplinary studies that centre women's and midwives’ spatial practices and experiences remain limited. Most research focuses on outcomes rather than on how design affects clinical practice, despite evidence that space shapes midwifery care (43–45). Given that most births in high- and middle-income countries occur in hospital settings, the absence of detailed studies on how design impacts health and wellbeing is particularly concerning (24). A paradigm shift is needed, away from a mechanistic, institution-led model of design and towards woman-centred design, grounded in users' perceptions and informed by a rich, interdisciplinary evidence base (24, 25, 46).

## 3 Methodological recollections of a high-risk birth

The study adopts an autoethnographic, qualitative, narrative case study approach to explore the sociospatial dynamics of an unmedicated vaginal birth ‘outside guidance’ of a first twin in an obstetric-led hospital setting, categorised as high-risk. It is phenomenological and rhizomatic, affective and emergent, attending to spatial intensities (47), and draws on the lived, embodied spatial experience (48), which is vital for connecting architectural form to bodily presence in birth. Autoethnography is used as a rigorous research method, triangulated by interviews with my birth companions, rooted in lived experience and emotion (49). Since I, the author, am both researcher and birthing subject, the article draws on personal experience, hospital birth notes, and reflections from my partner Colin O'Sullivan, doula Becky Reed, and obstetrician Philippa Corson to construct a narrative birth story**.** V was not included in the interviews because she was well represented through her written notes, which offered more insight than her limited verbal interactions at the time. Richardson (50) encourages using writing itself as a site of meaning-making and theory. And birth stories, as Kohler Riessman (51) suggests, challenge dominant discourses of objectivity, embracing positionality and subjectivity as critical tools for inquiry.

Central to this methodology is the understanding that birth unfolds not just physiologically, but through space, affect, and relational practices: space as a social product, not just a backdrop (52). Birthspaces are lived, contested, and ideologically charged. The analysis extends through the production and interpretation of architectural drawings and photographs, mapping how spatial elements, such as room layout, materiality, thresholds, and visibility, interact with emotional states and caregiving practices, exploring the interaction between institutional power and embodied experience (53). This multi-modal methodology highlights the agency of space in shaping experience and enables a layered exploration of design, embodiment, and institutional power. The chronological story of the day traces intensities and spatial transitions as they emerged in the narratives of the three participants. In doing so, the birth story becomes not only a mode of knowledge production but also a spatial critique and an act of reclaiming childbirth as situated, relational, and embodied.

### 3.1 Sociospatial theoretical framings

There are three spatial theories which are central to the study: space syntax (54), birth territory (22), and depth structure (55, 56). Space syntax quantifies spatial relationships, assessing how well-connected or segregated a space is. Key measures include ‘integration’ (how easily a space can be reached from all other spaces within a spatial system), step depth (number of spaces, threholds or ‘steps’ passed-through to reach an area), and visibility (how much of a spatial layout can be seen from any given point) (54, 57, 58). Widely applied in hospital design, space syntax helps researchers understand how spatial configurations shape behaviour, communication, and social engagement (58, 59). Spatial arrangement influences wayfinding, privacy, security, staff–patient interaction, and efficiency (50), and low integration can improve privacy by reducing unnecessary movement and visibility (60), while higher integration can enhance collaboration between caregivers, improving patient safety (58). In maternity settings, birth rooms with high integration were associated with better care satisfaction but lower satisfaction with privacy (60).

In birth territory theory, Fahy and Parratt (22) suggest birth environments exist on a spectrum between ‘surveillance’ and ‘sanctum'. The sanctum is quiet, private, its boundaries protective rather than restrictive. Here, the philosophy is one of trusting the physiological process of birth and safeguarding autonomy (integrative power). The surveillance room is a clinical space where the hospital bed takes centre stage, designed around observation, where birthing people are positioned more as patients to be managed than as active participants in their own experience (disintegrative power). Goldkuhl et al. (24) explored surveillance and sanctuary in different hospital rooms in Sweden and found that they are created not only by architecture but also by how caregivers behave. They call a birth well-supported by caregivers in which the person giving birth experiences a sense of sanctuary and autonomy the ‘personal room’ and contrast this with the ‘institutional room’ in which the birthing person feels subjected to the rules of the institution and these take precedence over her autonomy.

Depth structure (55, 56) is a conceptualisation of embedded social space which brings together the space syntax theory of integration, step depth, and visibility (54, 57, 58) with the sociospatial theory of Lefebvre and Soja (7, 52, 53). A depth structure is a series of spatial zones which divide up a room or building, each of which has its own set of social norms, or decorum. The zones are defined by physical features, which form thresholds and define where decorum changes. Usually, the zones closest to common-to-all places (such as the street or corridor), with the least step depth in space syntax terms, tend to have decorum which is more public in character, the zones deeper into the structure tend to have more private characteristics, and the decorum may be more specific or exclusionary. The idea of depth structure led me to purchase the cloth to create a new zone in the room in the first place, as well as informing my understanding of what happened on the day I gave birth*.*

## 4 The story of the birth of Julian

This is the story of the birth of Twin 1, Julian. Twin 2, Críostóir, was born about an hour later by Category 1 emergency C-section after a failed breech extraction, which, while urgent, I did not find traumatic. As I laboured with the twins, people (and babies) moved from here to there; we occupied a sequence of spaces in different ways. The chemicals my brain released mean my recollections are not linear, yet the physical memory of the spaces I occupied and the details of each are etched into my mind. The following story was woven together from the narratives of the three people I interviewed, along with excerpts from the official birth notes (italics) alongside my own recollections.

### 4.1 The background to this birth

Due to the high-risk nature of my pregnancy and birth, I was not eligible to occupy the environment designed for woman-centred care and optimise conditions for physiological birth: the birth centre. I had five biomedical risk factors that ‘place the mother and/or her baby at increased risk for adverse outcomes’ (61). In 2012, I had a traumatic first birth: an obstructed labour, a caesarian section under general anaesthetic and a severe haemorrhage which resulted in posttraumatic stress disorder and postpartum anxiety. I therefore had a non-standard T-shaped scar on my uterus for which very little research on vaginal birth after caesarean exists. I was also pregnant with twins; I was old (39); it was an IVF pregnancy. I'd had bleeding during pregnancy which had resulted in being admitted to the hospital twice, threatened early labour at 29 weeks, and then when labour began in earnest, it was premature at 32 weeks and 4 days. The guidance for the birth was a scheduled c-section, or at the very least, labouring with an epidural in place to facilitate fast relocation to theatre. I generally avoided telling anyone in a white coat my desire to have a much-longed-for vaginal birth, because it was exhausting having the same conversations repeatedly. I planned for it anyway, engaging a doula and considering the pros and cons of a home birth.

During the run-up to the birth at around 29 weeks, I was admitted to the hospital due to heavy bleeding and threatened labour. I spent two nights in the room where I eventually gave birth, so it was familiar territory. I also briefly met Philippa during this admission, explained about the previous birth, PTSD and other factors, and both my Colin and I liked and trusted her immediately. When I went into labour, due to the prematurity, it was essential for the safety of my babies to be near high-level neonatal medical care. Fervent voices argue that woman-centred care is especially essential for those who find themselves vulnerable or marginalised (66). For people like me, however, who must birth in a highly medicalised environment, ‘woman-centred’ care is often a distant pipe dream. Nonetheless, I had a safe, satisfying birth.

### 4.2 The birth environment

**Figure 2:**
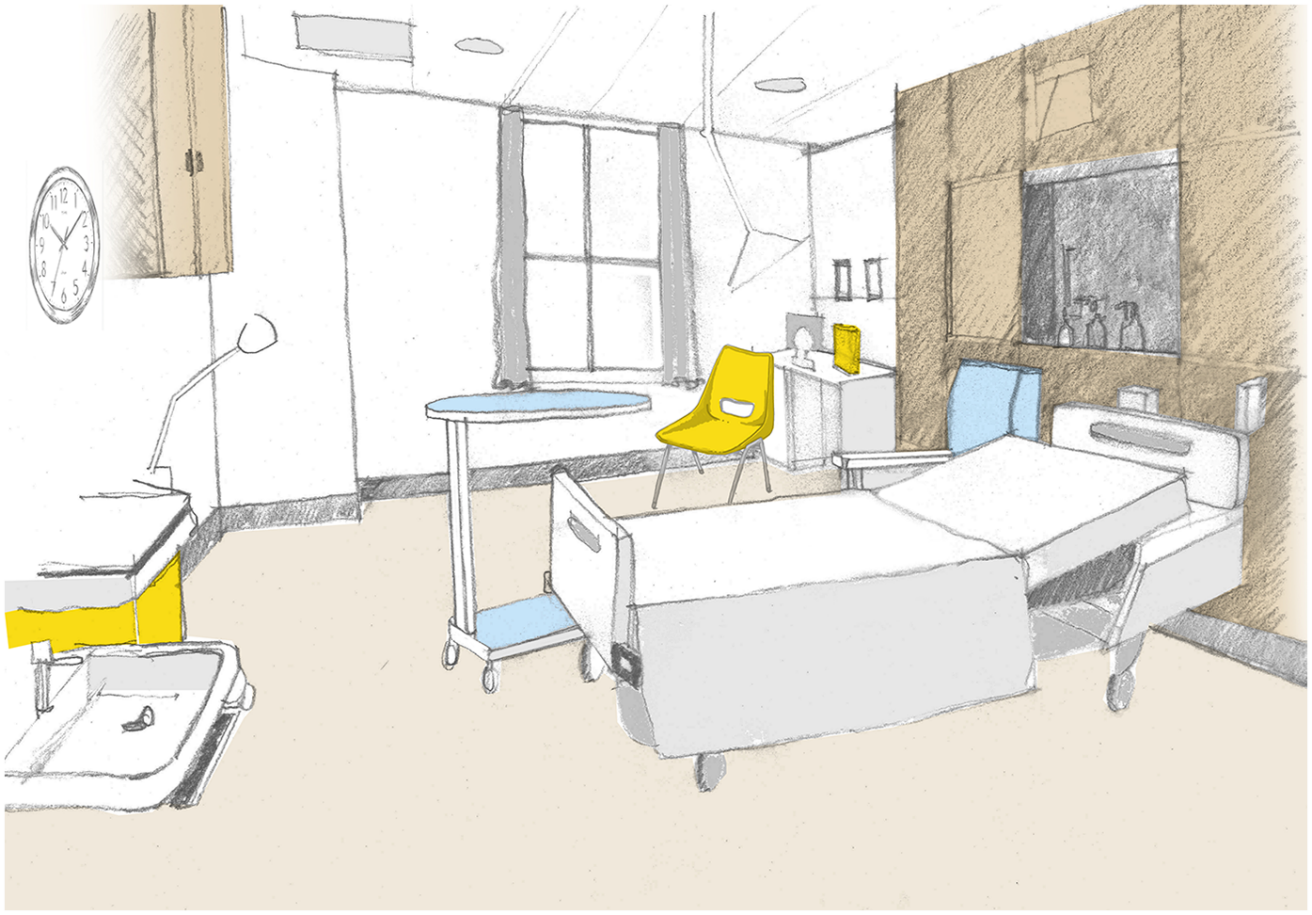
Drawing of how the birth room at the Royal London Hospital was when we entered, before we erected the cloth screen. Drawing by the author.

I laboured in a room at the high-risk end of the labour ward, a drawing of which is shown in Figure 2. Central to the room was the bed, positioned crossways and flanked by a locker for personal items, a visitor's chair with dark blue plastic upholstery, and a wheeled table for meals. Opposite the bed hung a large institutional clock. Above, a ceiling-mounted examination light. The room, approximately 9 m  × 5 m, had a corridor door at one end and an ensuite bathroom door set at a 45° angle. A beige curtain screened the corridor door. At the far end, a tall window looked over the city, its cream curtains ineffective at blocking light. Off-white walls displayed A4 hygiene posters, and the ceiling featured grey tiles, lights, sprinklers, and vents. Along the side of the room, timber-effect cupboards concealed oxygen, gas and air, and electricity supplies, attempting a more domestic appearance. Near the window stood a desk with a PC and a yellow stacking chair. One wall held a sink, a yellow bin, and dispensers for gloves, aprons, soap, and towels. The 2 m  × 2 m bathroom had grey–blue decor, a shower with grab rails, a toilet with a black seat, a sink, a mirror, and a wheeled shower chair. A blue exercise mat was rolled up in the corner, along with two cribs containing towels and, later, resuscitation stations.

### 4.3 Arrival and triage (space: the triage room)

Having been awoken by strong twinges at 1 a.m., I arrived at the Royal London Hospital around 2 a.m., was briefly examined by a midwife, and laboured in a triage room for around 2 h, mostly without a caregiver present. Wearing a soft yellow t-shirt and a pair of pants, I stood and leaned on the bed, humming through the contractions. Colin went out repeatedly to inform the staff that I was in labour, and I was surprised they did not deal with me sooner, but there was a sense that the place was very busy.

I remember the sunrise being beautiful, but the room we were in—when we saw the sunrise—was facing the sunrise. (Colin)

*4.50—Reviewed by doctor, vaginal examination (VE) shows 3 cm dilated, partially effaced, declined monitoring. Explained in view of foetal tachycardia pros* *+* *cons of monitoring, agrees to have CTG. In view of previous complications, understands risk of scar/uterine incision, C-section declined. Analgesics offered.*

I was eventually attached to a foetal heart rate monitor, lying on my back, and it was agreed that I was in established labour with regular contractions. The babies' heart rates remained within normal range throughout labour and did not decelerate.

### 4.4 Transfer to labour ward, first stage labour (places: screened birthspace and bed)

I walked with a midwife to the high-risk labour ward room.

*5.30—Admitted and transferred to labour ward, but no midwife available to take over care, awaiting labour ward midwife to take over care. Registrar did bedside ultrasound to determine position of babies (one head down, one breech), client cannulated by anesthetist.*

**Figure 3:**
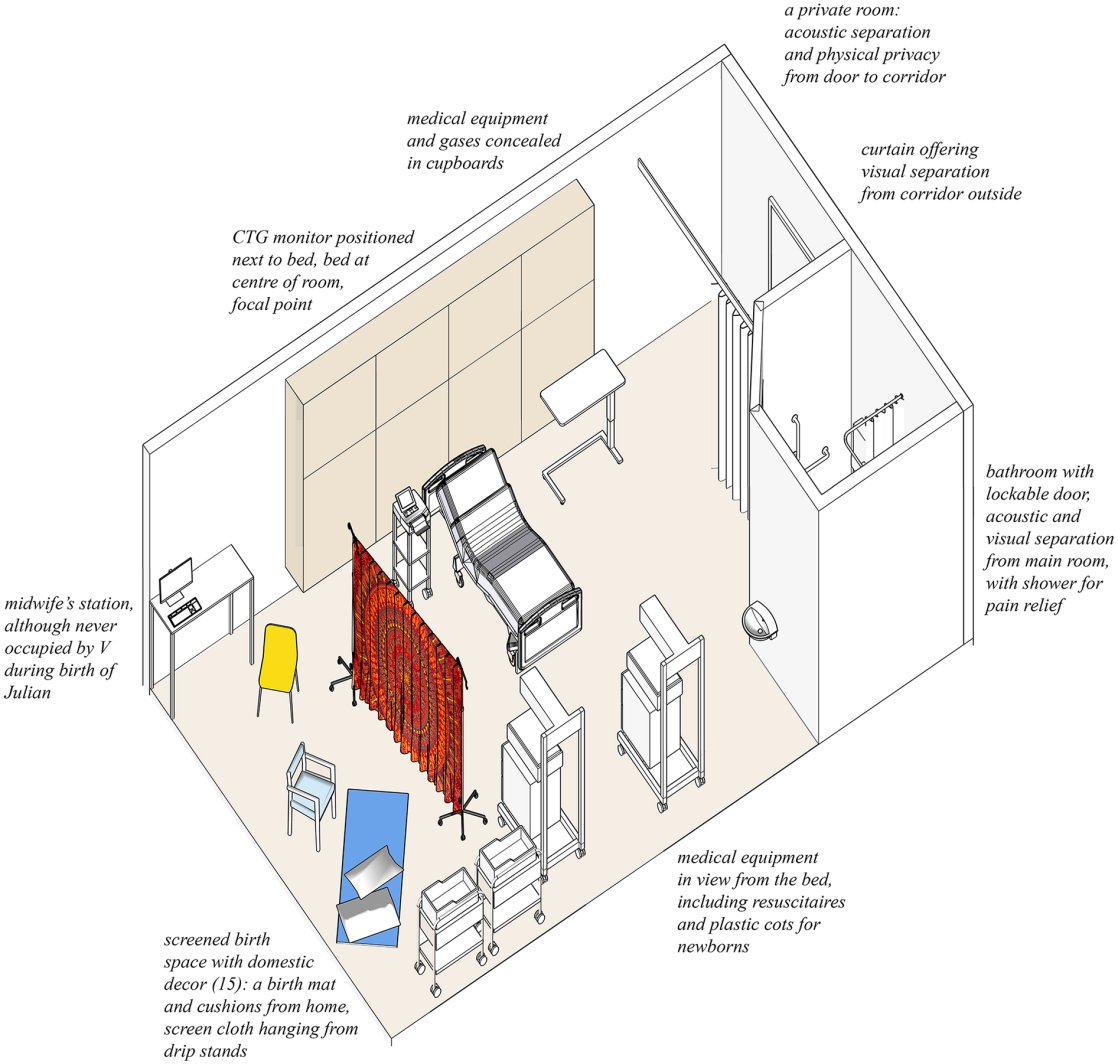
Axonometric drawing of the birth room at the Royal London Hospital, a high-risk room on the labour ward room. The cloth is shown attached to drip stands, creating a screen to divide the room, creating a new zone in the depth structure. Drawing by the author.

On my instructions, Colin set up the screen. We hung the cloth from drip stands across the room about two-thirds of the way between the door and the window. It was dark red, printed with a circular mandala-style design which reminded me of a uterus (see Figure 1). It created a saggy screen, and behind it, we put the visitors' chair and the exercise mat on the floor. Unlike everything else in the room, I had designed and made this space for myself. An axonometric drawing of the room including the screened birthspace is shown in Figure 3.

I remember clipping it to stuff or trying to find ways to clip it to things. I remember those metal pegs in the box with the little rubber ends on them … I do remember trying to resist the temptation to string it onto a really important machine. I think they were all looking at me thinking what the f*ck are you doing? I didn’t really care. (Colin)

I remember the screen … I remember it being very set up, in the way that you wanted. And that was good, I think. I had a woman … having a vaginal birth after cesarean. And she had put fairy lights and things around the bed. And I remember walking in and just, she looked at me and I looked at her and I said, “I can see how much you really want this birth”. And she was just like, yes, yes, I want it so much. And sometimes, that desire to have that space is also kind of representative, I think, of how much you want a certain thing to happen … And actually, again, some of my colleagues don’t like it. But I quite like it when somebody has made a space theirs. And taking the time to encourage somebody to feel comfortable with you, at home with you, safe in a room in an environment that there's a two-way thing there, and they have some power and control. (Philippa)

A midwife asked me to get onto the bed for further monitoring and a VE.

*6.35—Midwife takes over care. VE shows 4 cm of cervix. Client vomited and offered anti-emetic. Client is lying on the floor mat and used a cloth to divide rest of the room upon arrival.*

6.42*—Client declined an anti-emetic; auscultated babies [listened to heartbeat sounds] and took blood pressure.*

The monitoring was very time-consuming as there needed to be two belts, and they kept slipping, or picking up my heart rate. Every time they slipped, the monitoring period would restart.

*6.45—CTG monitoring session: noticed that trace might be picking up the same fetal heart rate. Will ask the doctor to come and scan client.*

*6.51—Doctor scanning client and has picked up both foetal heart rates.*

*7.03—Client changed position onto bed, and it’s difficult to auscultate.*

I do remember those belts, though … it couldn’t ever stay on, like, it kept moving. And I was looking and thinking, in what f*cking medical environment do they think this is a good idea, and why can’t somebody invent a better one, like, where it's taped to you or something? Like, it kept moving. And then they’d go, “Oh, we’re not getting a clear reading”. (Colin)

*7.10—Client needing the toilet. Client said to have her off the CTG monitor immediately, which she did. Doctor has signed the CTG tracing.*

*7.20—Client is out from the toilet and is on the birthing ball currently.*

*7.30—Shift hand over notes: intermittent monitoring, [will] attempt vaginal delivery, anti-emetic, and analgesia [incorrect, I'd had neither], drink only clear fluids [in case of CS].*

*8.00—Client is in the shower currently.*

*8.30—Care handed over to midwife V. Plan: routine care.*

*8.43—(V's handwriting)—Jane in a partitioned area of the room. Call to doula Becky, she is keen to come and support. Agreed with coordinator she can come.*

Around this time, I had an unwelcome intrusion behind the screen. Hearing me groaning, a clinician offered unsolicited sympathy and pain relief. I interpreted this as a misreading of my experience, for I had not lost control and was embracing the process and was frustrated at having my vocalisations pathologised.

*9.00—Jane on birth mat, membranes intact, twins' heartrates monitored.*

*9.30—On birth mat, discussed need for VE to check progress. Phone call to neonatal SHO to inform of labour 32* *+* *4 twins.*

### 4.5 Agreeing to a C-section (place: bed)

*9.50—Discussion with patient and partner following VE findings* … *c/s vs labour, patient and partner will discuss.*

While it is not clearly written in the notes, I remember that at this VE, dilation was 6 cm. Lying on the bed for the examination was disempowering and uncomfortable, and I remained sitting on the bed while the recommendation was persuasively made to have a caesarean. Several staff had arrived to discuss options with me; the notes name four people plus the anaesthetic team. I vividly remember the on-call obstetrician saying I was ‘only’ 6 cm dilated and that ‘in my position she would have a CS’. They stood in a row of concerned faces, above me. I, by this point feeling powerless, agreed. I had fought institutional pressure for almost 7 h and explained myself repeatedly, while labouring, and my fight was gone.

I remember it as almost like a cinematic thing … The mental snapshot I have is you were on the bed in a room that it wasn’t so full of people, and then suddenly the room being quite full of people … like a herd of people … I remember feeling like it didn't belong to us anymore, the room … because when you were in bed every time somebody came into the room, no matter how junior they were, they were more expert than you and me in the view of the room. (Colin)

### 4.6 Philippa takes over and Becky arrives (place: bathroom)

Colin and I then escaped into the bathroom. I needed to pee after lying down for so long. I wanted to get into the hot shower, and most of all, I wanted to be away from surveillance.

And then everybody else was outside, and it was just a horrible bathroom, obviously designed by a contractor for the NHS, but it belonged to us, and there was nobody in there, and people had to knock to come in. It was peaceful … It was respite after all the insistence that you might be doing something wrong and putting your children in jeopardy … And then when the door was closed … even the most annoying medics trying to invade your privacy, they didn’t anymore because you could have been having a sh*t in the bathroom! (Colin)

At that moment, two things happened to change the course of events. Becky, my doula, arrived at the hospital. And, consultant Philippa, the Royal London Obstetric Lead who has a special interest in breech birth, took over my care and made decisions which supported my desire to birth ‘outside guidance’. Philippa knew my desires, because I had spoken to her prior to the birth. We had a good but brief meeting when I was admitted for bleeding; she was the reason I relocated my care to the Royal London from Guy's and St Thomas' at around 29 weeks.

I don’t think that I was the consultant on call that day. I think it was one of my colleagues, who was much more uncomfortable with what was happening … I had come in just after you’d had an examination by my colleague who’d said you should have a caesarean … And I think your partner was just like, “But she doesn’t want it”. And it just felt at that moment that it was probably right to say, “All right, you go, and do what else is going on in labour ward, and I'll stay here for a bit, just to see which way the wind is blowing”. And it was fortunate that day I was able to do that. But it seemed to me that was the right thing to do in those circumstances. (Philippa)

I think yours and my prior knowledge of each other … I trusted that you would listen to me, if the risk was too much. So, a two-way trust process, I think, with birth outside guidance … I am happy to hold a degree of risk that perhaps others aren’t always comfortable with, particularly if there's been a prior relationship … a lot of obstetricians will ask the question of “What if somebody wants to do something that's unsafe?” That word comes up quite a lot. “Have you told them it's unsafe?” … But there's a line to walk between ensuring safety and inflicting psychological trauma. That's my perspective.

I was met with what I felt like quite a shocking scene because I was arriving there to support you in your labour. And when I walked into the room, there was a bed with nobody on it. And Colin was standing by the bed and he said something like, “She's six centimetres and she's decided to have a caesarean”. And me thinking, what? Why? … I was absolutely clear in my mind that you had been absolutely clear in your mind that that was not what you wanted … Something must’ve happened that's made Jane very scared or there's been a terrible power story where she's been completely subjugated and this feels like the only way out, or the way through … I obviously straight away went to see you in the shower and you were just looking amazing and beautiful and comfortable … So then I was more confused … I mean, perhaps my position in that room then was actually just to look at you and say: “I am an experienced midwife. I believe you can do this. In fact, I believe you are doing it”. (Becky)

Becky did a couple of things to help me feel safe and contained, creating better conditions for physiological birth: she switched off the light in the bathroom, and she cheered me on, said how wonderfully I was doing, how normal everything seemed. She gave me some clear facts about the progression of labour and suggested that Philippa come into the bathroom to speak to me. Philippa spoke to me quietly one-to-one in the steamy darkness, as I had hot water pumping from the shower into my back. She reassured me that she saw no reason why I would be unable to have a vaginal birth.

Everything changed when [Becky] arrived. Like everything. You were different when she got there. And I think it must have been to do with the feeling of kind of protection, a bit, from all the brouhaha. And she changed and affected the timing of Julian's birth, because up to that point, they were beginning to be a bit like, “This needs to happen”. And people stopped talking about timelines as immediate as they had been the five minutes before she got there. (Colin)

I remember [Philippa] then being quite surprising because I was expecting, you know, “Okay, that's it, that's what we’re doing, we’re going for section”, and she said something like, “We’re busy at the moment in theatre, you’re doing fine, you carry on, you get on with what you’re doing, and I will come back and let you know when the theatres are free”. And at that moment, my message that I felt from her was, I feel you’re okay, I feel you’re safe, I’m not worried about you … And she then disappeared. I think she waited a little while and watched you have a couple of contractions; she then went off. (Becky)

*10.44 (Philippa's handwriting)—patient in shower; doula present also; patient feels progress is slow, worried about scar rupture and progression to emergency CS, warned that length of labour will increase chance of scar rupture; explained that there is another patient in theatre at present, prior to moving to theatre can reexamine to see if any more progress; patient happy with this; MW to kindly listen to foetal heart rate while patient in shower; continuous CTG after this.*

*11.10 (V's handwriting)—Jane in shower, doula present.*

### 4.7 Established labour (places: screened birthspace and bathroom)

**Figure 4:**
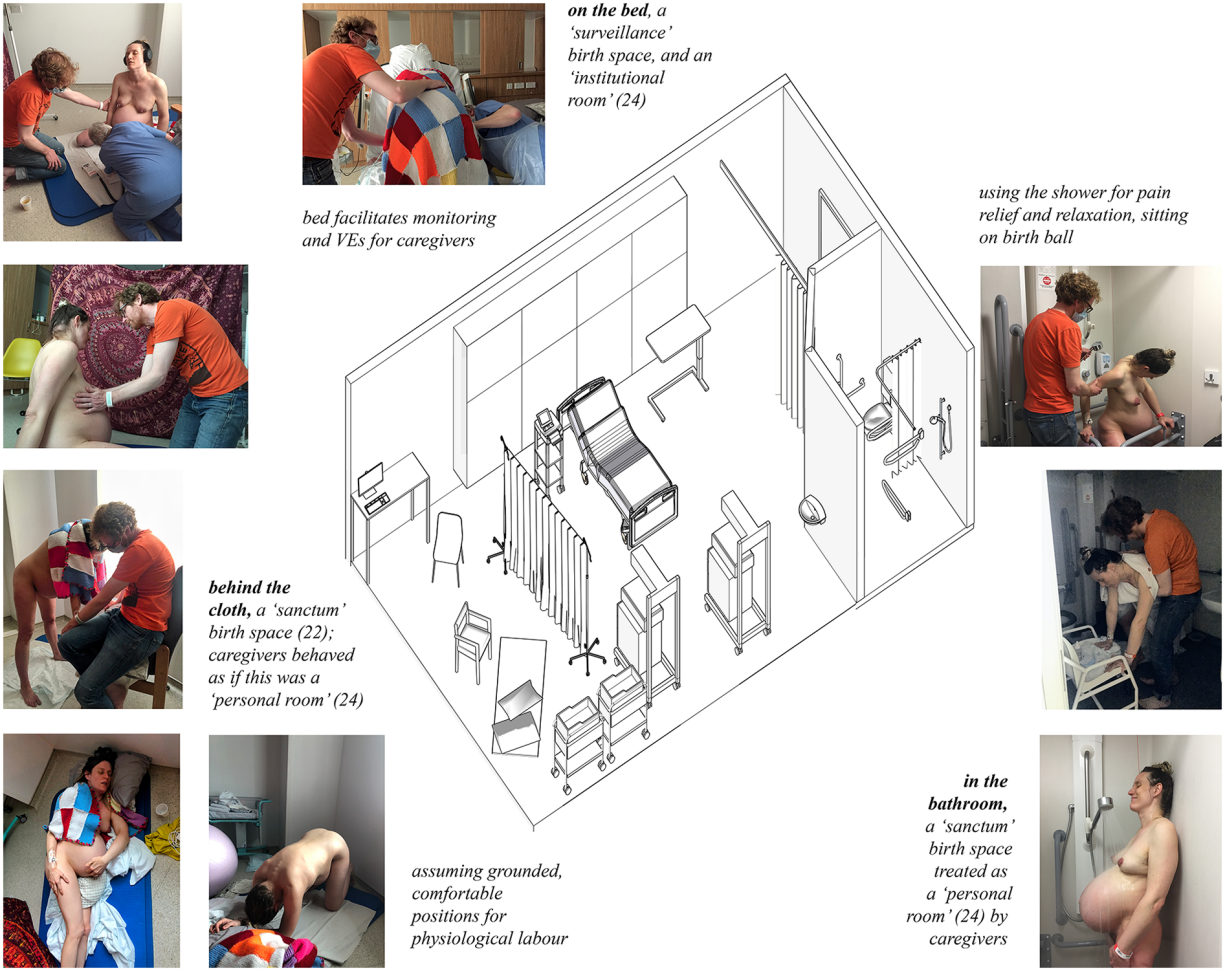
Occupation of the different zones in the birth room. Left, behind the cloth, where I moved between chair, mat, birth stool and floor. Centre, the bed, where I was monitored and given VE's. Right, the bathroom, where I stood under the shower in the private darkness. Drawing by the author and photographs by Becky Reed, used with permission.

As I gradually got deeper into labour, as the baby descended, I withdrew into an internal mental world. This was reflected in my choice of spaces, I moved from the bathroom where I stood under the hot shower which massaged my back and relieved the pain, to behind the cloth on the floor, or sitting on the birth ball which also moved with me. I leaned on the visitors' chair, the shower chair and the bathroom grab rails. I hung off Colin and the door frame of the bathroom. The sequence of spaces and how I occupied them is shown in Figure 4.

You were, in my experience … a woman in entirely normal labour, behaving normally. So just my usual behaviour with a woman in labour, which would be to say, you’re doing really well, this is great, you’re doing fine, you’re doing well. And you were showering yourself and having, yeah, in normal labour. And it sounded like it was progressing really well. (Becky)

*11.35 a.m.—On birthing mat, passed urine.*

*12.10 p.m. (Philippa's handwriting)—returned to reexamine patient prior to consideration of proceeding to CS; discussed options as now fully dilated, patient happy to proceed with vag delivery; discussed that we would advise to foetal monitoring at this stage ideally continuous CTG, other option is for auscultation every 5 min as now in active labour, patient declining CTG.*

Philippa said something along the lines of “You’re doing fine. What do you want to do?” And then maybe we had a little discussion or something, but it made absolute sense for you to carry on … then left. She left and I was completely stunned … And then you kind of went … “OK, that's it. I’m carrying on. Yes”. Sort of a decision made by you. Instead of saying, “Oh, I’m defeated, I’m going to theatre, that's it”, you kind of went, “Yeah, actually, I’m doing this”. (Becky)

I went hot and had to throw everything off, and then cold and went under the shower or wrapped myself in my special blankets (Figure 4). I was in my own little bubble of intense concentration: noise-cancelling headphones, eyes closed against the room, which was too bright thanks to the light curtains, cracking them open only briefly to make eye contact with Colin and Becky for reassurance. At times, I needed to leave my sanctuaries. When requested to do so, I went on to the bed, for a vaginal examination or a period of foetal monitoring on the machine, although as labour progressed, this stopped and the midwife V shifted to intermittent mobile monitoring with a handheld Doppler, as staying still on my back was impossible.

### 4.8 Second stage (place: screened birthspace)

*13.00 (V's handwriting)—On mat, Becky giving excellent support.*

Once Philippa had declared herself to be content with the situation and gone away, it seemed to me that the midwife then thought, “I’m not going to worry because the consultant's been in and said we’re OK”. … This is not what I was expecting … no doctors came in after that until Philippa came in later. I don’t remember people walking in. What I remember is very few people coming in, and my amazement of being left to get on with it. (Becky)

*13.10—Remains fully upright, neonatal team ready nearby.*

You were being a woman who was behaving instinctively … And very active and very upright. I knew by then that you knew that you could do it. So you were never at all, as far as I can remember, doubting yourself. In fact, you were slightly unusual because most women do. But you were not saying “I can’t do this”. You were saying “I am doing this”. So, something had given you that strength and that awareness and that belief … It all felt very straightforward. (Becky)

**Figure 5:**
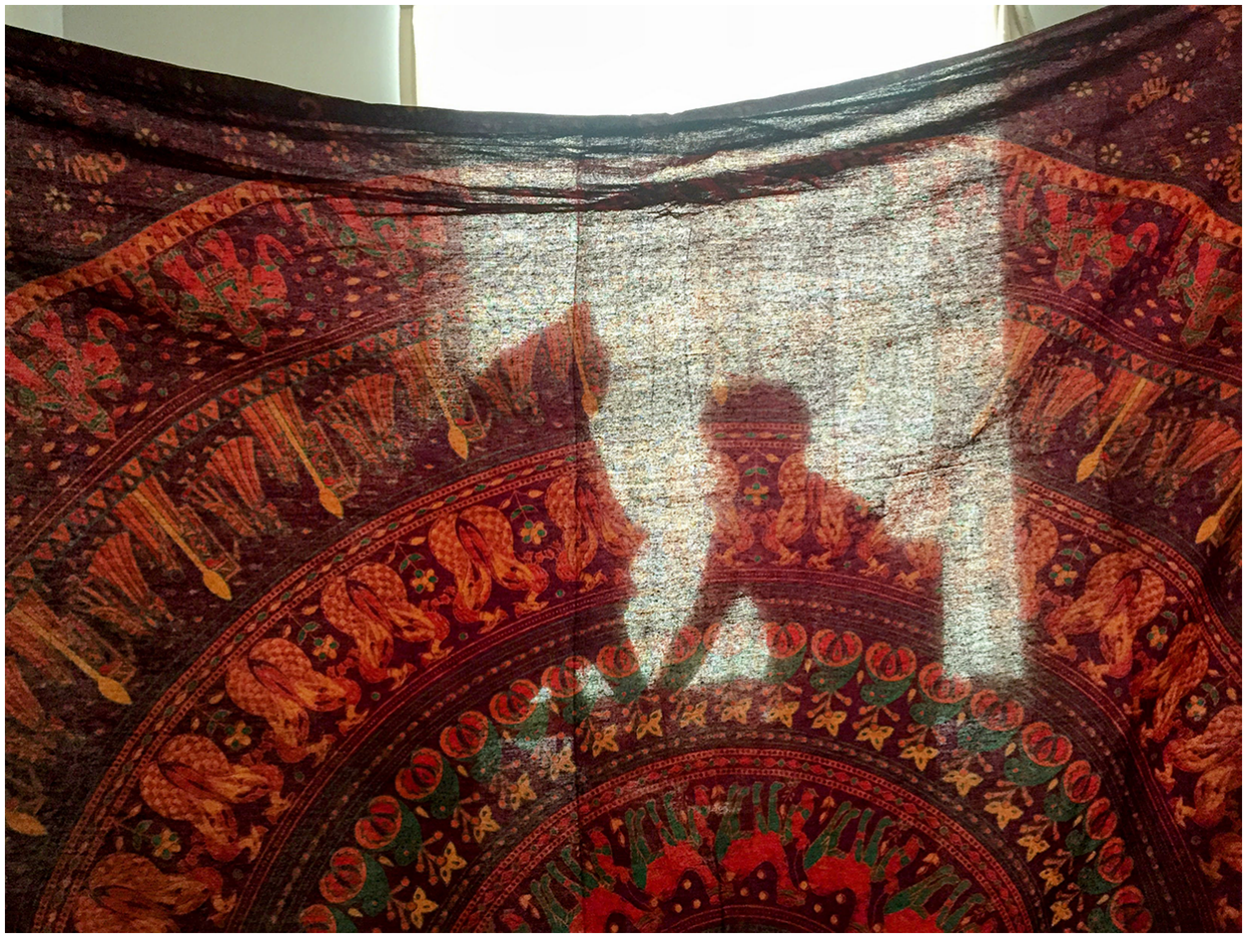
Colin and I behind the cloth across the birth room, as I laboured. This photo was taken at about 13:00. Photograph by Becky Reed, used with permission.

By the second stage, I was ensconced behind the cloth (Figure 5 shows a silhouette of Colin and I behind the screen). The light was bright from the window, and the reds, purples, and deep oranges on the fabric framed a backdrop which contained my trusted people.

That space that we created behind the cloth—what was it like? Surprisingly roomy. Very safe. Quite excluding. Very lovely colours … that was the effect of the effect of the of the cloth you had used, which was calming. But the main thing really about it was the separation, that it separated you from the hospital environment, and the bit of you that had agreed to the cesarean had gone. It was on the other side of the curtain … It separated that person from the person who was going to get on with it and do it. (Becky)

*13.45—Standing and squatting, coping very well with pain without analgesia.*

And then being behind the curtain. I remember being over near the window then, and I remember the red leatherette chair. Maybe it was blue. It was red or blue. Something was red and something was blue. I remember you sitting in that chair … I remember draping things around your shoulders. I remember looking out at the sun from time to time. The jeans I was wearing, a red t-shirt, maybe. I remember holding on to you. At some stage, I was sitting down, and I was holding you. You were facing that way as well, and you were hovering over the floor. (Colin)

### 4.9 The birth (place: screened birthspace)

Philippa mentioned that I was nearing the end of my allotted 2 h time for pushing. I had a sense of panic when I heard that, because in my previous labour, I ‘ran out of time’, and it resulted in interventions which I was desperate to avoid. So I pushed mightily.

*13.55—Urge to push.*

I think you were squatting, kneeling back, leaning back against Colin. And then I think I think I got you sitting on a bedpan and you were pushing. (Becky)

*14.30—Philippa ruptures membranes.*

You were walking around about the time that you felt the urge to push. And I was literally crawling after you on my hands and knees because I was worried that this obviously small early baby would come out suddenly and plummet to the floor. And then  … you stopped to be sick, and I was frantically crawling in the opposite direction so it didn't get in my hair. Whilst also thinking, “I mustn’t let this distract me from, the possibility that this baby is quite imminent. (Philippa)

*14.47—Bedside (sic) ultrasound scan to check position of second twin.*

So there was these two young women with different monitor machines … They were like scientists or something coming to do a test. It was funny, because it's very, I don’t know, this very millennial old thing was happening on the floor, in a very on-the-floor kind of way, you know, because you couldn’t see the hospital beds, and you couldn’t see the machines that go ping, and you couldn’t see [the women] either. (Colin)

*14.58—Turned, facing forwards, standing.*

Every time somebody [came around the cloth], you got a glimpse into the room, and because I was sitting right next to you, and I was very concentrated on your breathing and your guttural noises. I would notice people coming in and out of the room, and so sometimes you’d look and there’d be like two people, and then there’d be 15 people. There was a ridiculous number of people in the room. (Colin)

At some point also quite an army of neonatologists came in as well, but we ignored them … Let's not look at them at all because we don't need them at the moment. (Becky)

*15.00—Pushing.*

I guess it's rare for that kind of thing, for somebody like you to have a vaginal birth after having such a traumatic first [birth], then with twins, and the fact that they’re early … It did feel like we were celebrities … And there was people, like, whispering and, you know, talking about you, it was a very intense time. And just before he was born, sh*tloads of people came into the room, and it was like being the guinea pig, or the test case scenario … You can imagine people, like, all the way down the hall, it's happening now, it's happening now. (Colin)

*15.04—Baby born.*

**Figure 6:**
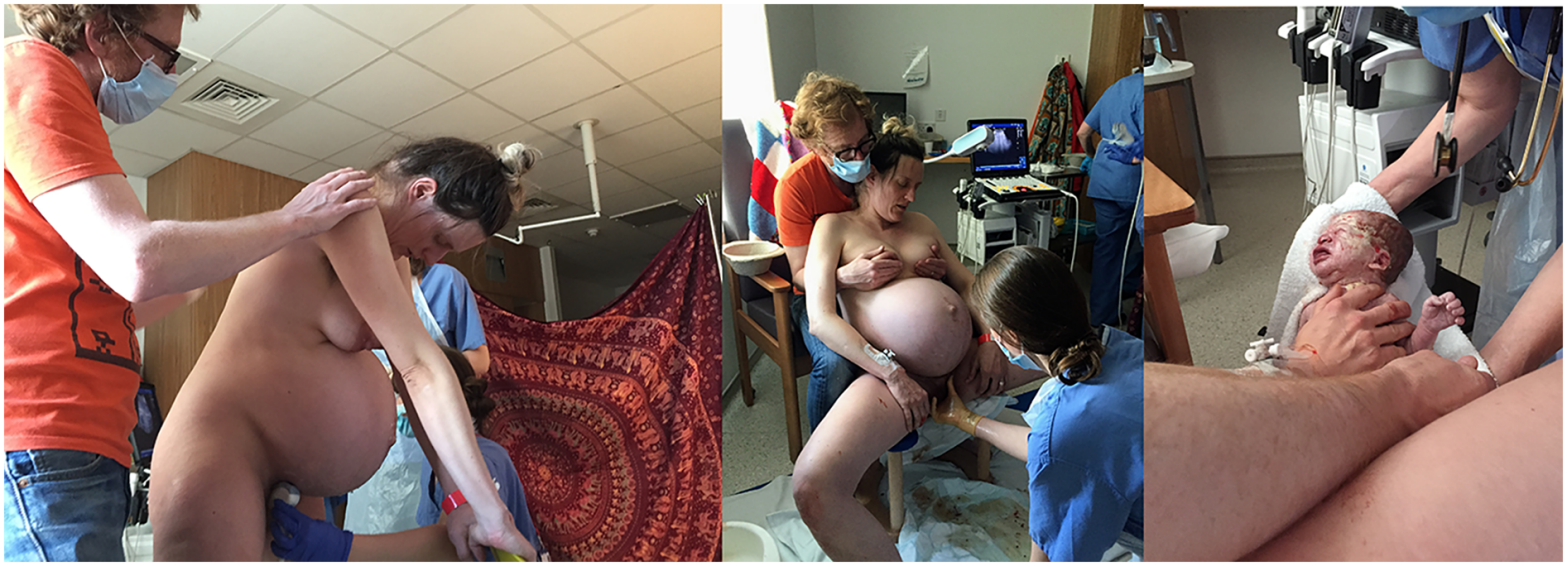
The moments before and after Julian's birth in the screened birth space with Philippa, Becky, V, and Colin present. Left, leaning on the chair, supported by Colin. Centre, me on the birthing stool, having just had my waters broken by Philippa, held in a ‘bound’ position (62) by Colin. Right, the moment of Julian's birth as I immediately reached out to touch him. Photographs by Becky Reed, used with permission.

**Figure 7:**
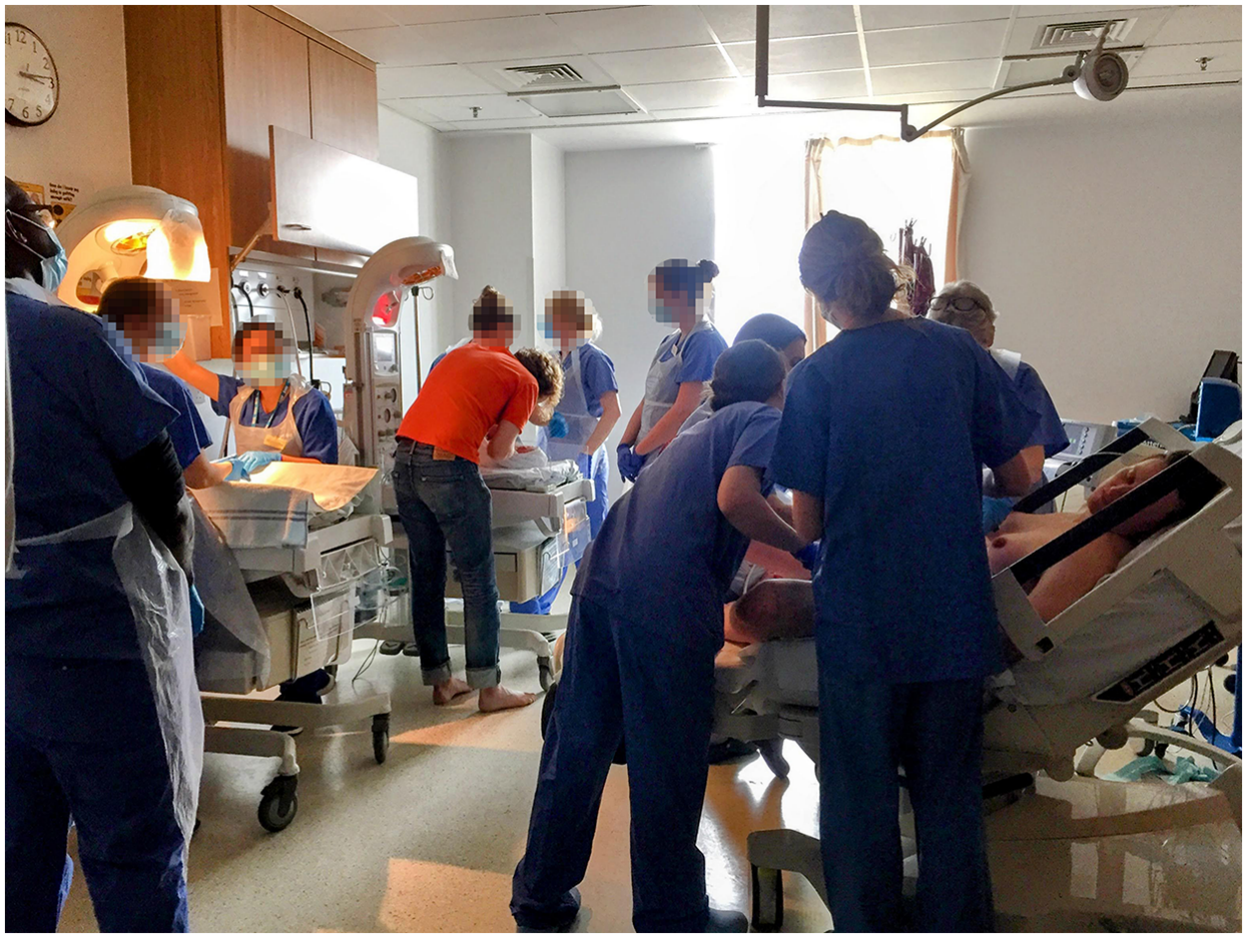
‘The herd’ of people occupying the room. Picture taken after Julian was born, I am on the bed while the obstetrician tries to turn Criostoir around, Julian is on the resuscitaire. There are 10 staff members in this picture. Photograph by Becky Reed, used with permission.

Julian was born behind the cloth. I pushed him out; it felt momentous and powerful; I was a whole person, with jurisdiction and agency. I did it, it was not done to me. I was crouching with my legs wide on a blue birth stool which V had sourced from somewhere. Colin was holding me up from behind as I hung off him. Behind the cloth were me, Colin, Phillipa, Becky, and V. Phillipa was kneeling in front of me, supporting my perineum as the baby's head was born. She caught Julian in a towel and handed him to me. Unlike lying on the bed, with the team above me, I was eye-level with her. Becky was also kneeling, holding a mirror so I could see him emerging from my vagina, and taking photos. Photographs of this sequence of events are shown in Figure 6. V was off to one side, supporting with whatever Phillipa needed. I could not see the ‘herd of people’ and ‘machines that go ping’ on the other side of the screen; these are shown in Figure 7.

And you pushed him out. It was, you know, as it should be. It was magical and amazing and straightforward and normal and expected by me, at least … I think for me as well, I felt completely safe … And it was all just a very, very straightforward baby birth … Your response is exactly what women do. “I’ve done it. I did it. I’ve fucking done it. My body's done it. I’m amazing” … And you were amazing and you did f*cking do it and it was incredible. (Becky)

I think my take home from it was that it was really very powerful … Julian came out, to be able to just sort of pass him through to you like that and see the expression. It was actually a very beautiful moment. It stuck with me. (Philippa)

## 5 Reflecting on birth in a nested depth structure

The birthing room should have different spaces which allow the woman to retreat, use the bath or toilet but maintain privacy when she chooses. The process and pain of labour and birth induces various responses and women try to withdraw, to find places where they can be undisturbed, preoccupied with their feelings and focus on the changes taking place as the birth progresses (9).

### 5.1 Privacy and protection in the birth environment

**Figure 8:**
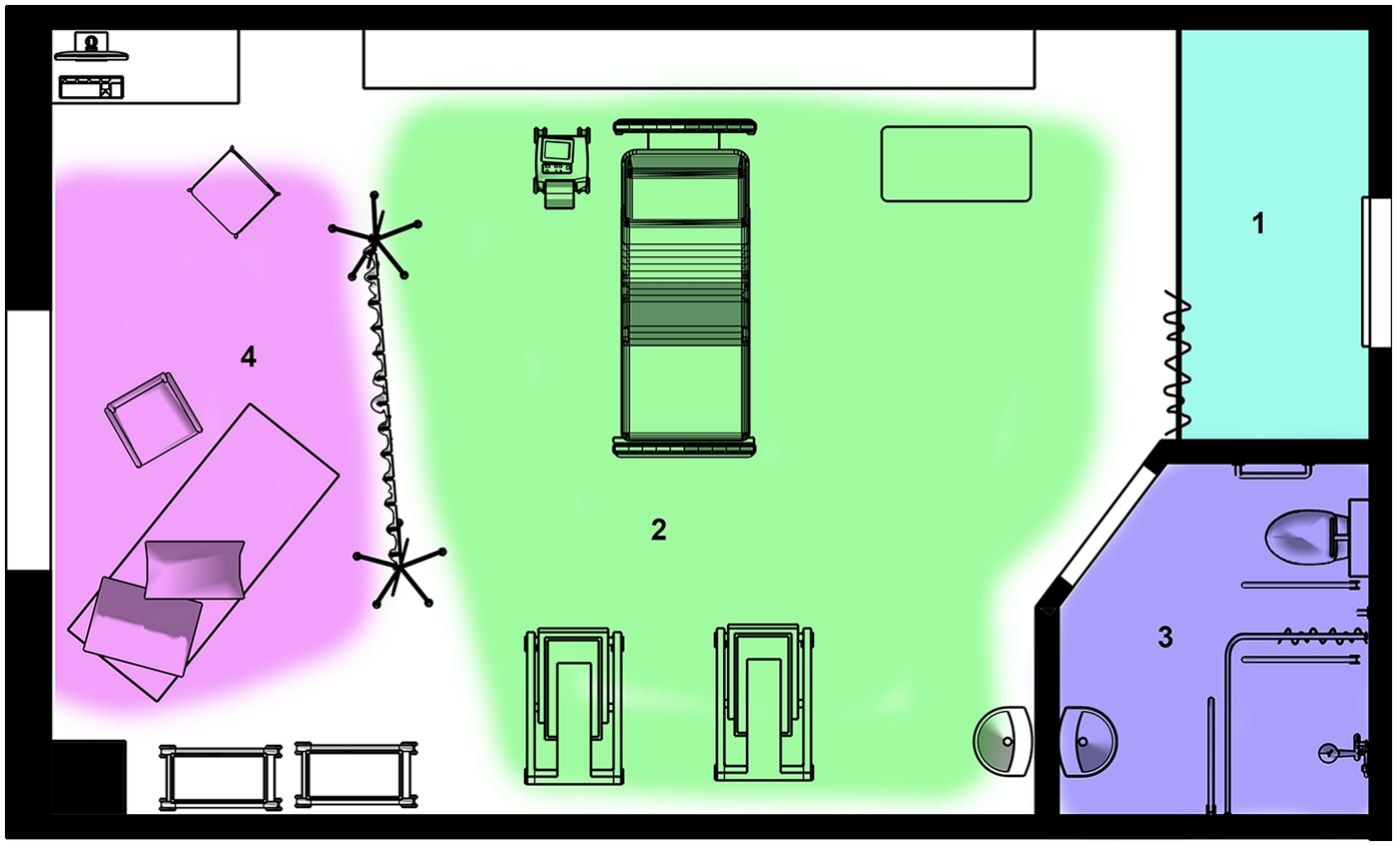
Zones in the depth structure of the birth room. (1) Behind the curtain separating the door from the rest of the room. (2) The central area containing the hospital bed. (3) The bathroom. (4) The new zone created by me, behind the cloth, the ‘screened birthspace’. Drawing by the author.

**Figure 9:**
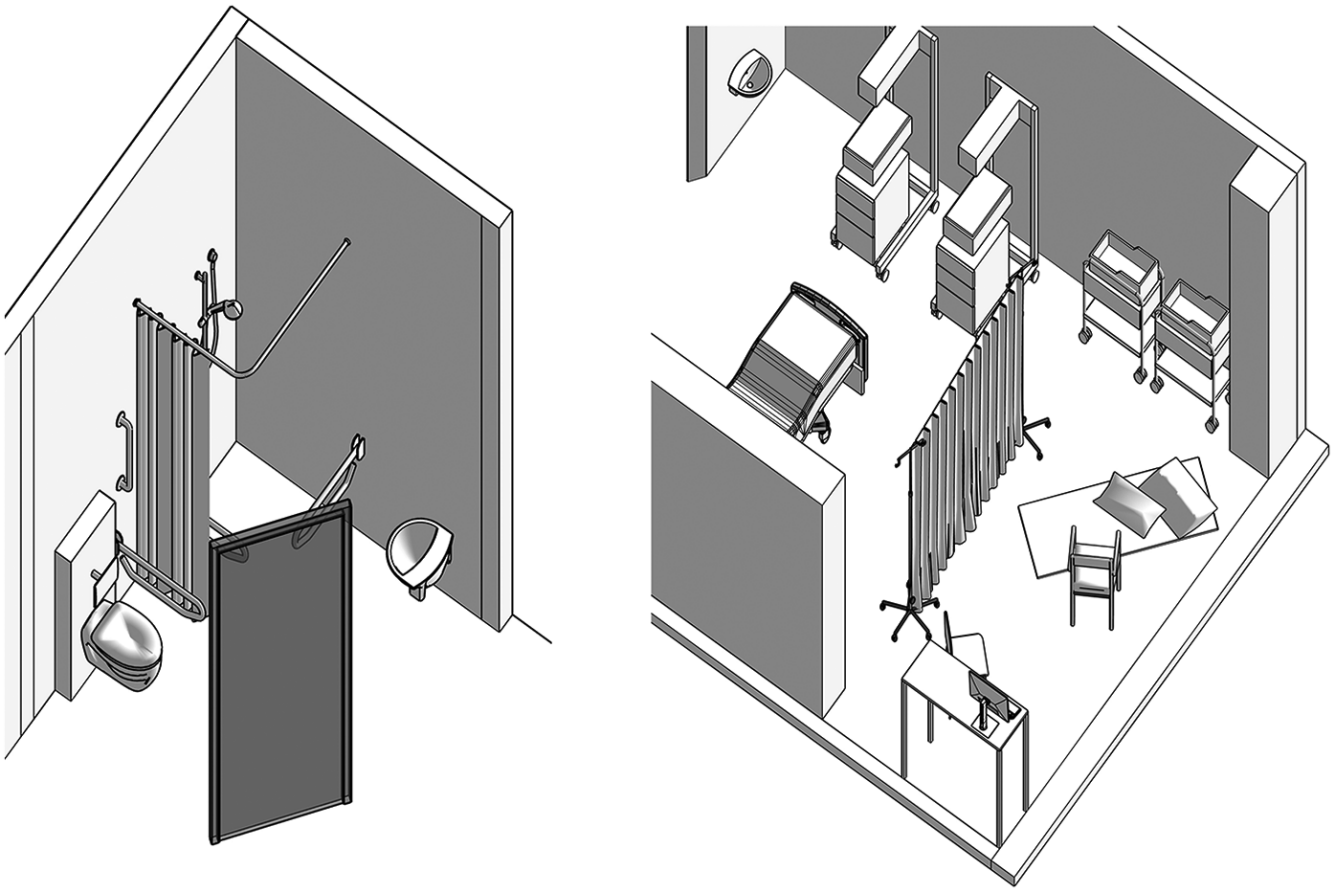
The least public zones in the depth structure of the birth environment. Left, the bathroom showing the shower, toilet, sink and grab rails. Right, the screened birthspace behind the cloth showing the chair, exercise mat and pillows on the floor.

After the screen was erected, the room had four distinct places, three of which were occupied at different times: the bed, with monitoring equipment; the bathroom, with a door which could be shut and locked; and behind the screen, the birthspace. These zones in the depth structure are shown in plan in Figure 8, and the most private of these, the bathroom and the screened birthspace, are shown in Figure 9. There was also the space between the door and the curtain although labour did not take place there as it was too close to the door, which served as an extra layer of privacy and screened the room from the corridor. Behind the screen, a combination of items already existing in the room (the birth mat and the chair) were utilised, as well as items brought from home (pillows, blankets, and the birth ball). Later, new things were brought by V: a bed pan and a birthing stool. Joyce calls the use of furniture already a birth room in new ways a spatial practice of ‘finding affordances’ (41), and across the literature, different authors condone the use of comfort items to make a space more familiar (10, 24). In terms of birth territories (22), the bathroom and the space behind the screen had more characteristics of ‘sanctum', and the bed was closer to ‘surveillance'. Table 1 shows a comparison of the characteristics of the different spaces used during the labour and birth experience.

**Table 1.** A comparison of the characteristics of the different spaces in the birth room, according to sensory conditions and relating to the three different sociospatial theoritcal framings discussed in section 3.1.

Conditions	Corridor-side curtain area	Central bed area	Bathroom	Screened birthspace
Physical Features	Door, beige curtain; closest to the corridor	Hospital bed, ceiling light, clock, medical equipment	Lockable door, shower, toilet, mirror, grey–blue interior	Fabric screen, exercise mat, chair, blankets from home
Acoustics/sound	Open to corridor noise, little acoustic protection	Exposed to noise and interruptions	Good sound insulation, acoustically private	Muted by the screen although still potentially load, cocooned atmosphere
Decor	Standard hospital decor, institutional	Institutional, clinical focus	Functional, cold-coloured NHS bathroom	Rich colour fabric, personal items from home
Lighting	Bright light from the corridor	Ceiling light, harsh lighting	Dimmable/controlled by user	Filtered light through the screen, natural light from the window shielded from the main ceiling lights
Threshold/boundary nature	Curtain only; visually semi-permeable	No physical boundary; open access from any side	Solid lockable door; clear social and physical boundary	Fabric screen; visual barrier, weak physical barrier, respected by all
Step depth, visibility, and integration (Space Syntax)	Low step depth, high visibility, low integration	Central location, high visibility, high integration	High step depth, low visibility, low integration	Added depth through screen, reduced visibility, lowered integration
Birth territory type	Surveillance	Surveillance	Sanctum	Emergent sanctum
Gatekeeping and decorum by caregivers	Minimal; curtain offers weak threshold	Controlled by staff (VE, CTG use)	High control by birthing person	Collaborative: respected by staff, gatekept by Becky and Philippa
Facilitation of SSB	Low: exposed, transitory, no privacy	Mixed: access to care but disempowering; triggered fear cascade	High: warm, private, enabled retreat and autonomy	Very high: sense of ownership, safety, reduced surveillance

### 5.2 Gatekeeping

Architecture and decor can only go so far when it comes to supporting a person to have a safe, satisfying birth. Of equal or perhaps greater importance is the caregiving received, and in this case, the gatekeeping and protection of the boundary created. In a talk she gave for a workshop for Spaces of Birth and Death, Phillippa described how the obstetrician oversees the scene, and everyone looks to her for cues about how to behave (63). Her taking over of my care and willingness to hold the risks of birth outside of guidance set the tone. During the birth of Julian, Philippa was respectful of the screening, and she joined me in my chosen place of the floor, a position Becky said she had almost never seen an obstetrician assume. Philippa co-created the decorum of the screened birthspace sanctum, guiding others in the room from her position of power. Because of the screen, alongside the collaborative gatekeeping of Becky and Philippa, the decorum which emerged was one that respected that screened sanctum boundary as real. It shaped the atmosphere and impacted the behaviour of all the caregivers present.

Becky's presence also co-created the decorum in and around the screened birthspace. She was my source of continuous emotional support. As Goldkuhl et al. (24) found in their study of birth environments, a familiar and adaptable physical environment does not determine a birthing person's agency. Instead, their experience is shaped by the support; agency is more closely linked to care practices and birth philosophy than to spatial design alone, particularly a culture of non-disturbance so a person can retain their ‘birthing consciousness’ (64). Decades of research have reinforced the positive impact of relationship-based care on birth outcomes and experiences (29); skilled birth companionship requires self-awareness, emotional regulation, and confidence in navigating complex situations (65). Key attributes include warmth, openness, sensitivity, and the ability to build strong relationships (62, 67), and these qualities positively shape childbirth experiences, with lasting emotional effects (68). Both Philippa and Becky were warm, open, and skilled at creating strong relationships, as well as sharing a philosophy of person-centred care. Becky, like the cloth, was from outside the institution and did not need to align with its goals, regulations, and guidelines. Philippa has a philosophy of establishing two-way trust with birthing people and is willing to bear the risks of supporting desires for birth outside guidance. Their combined expertise and experience worked together, neither competed for overt control nor attempted to exert disintegrative power.

### 5.3 Decorum in the screened birthspace

The existing depth structure (55, 56) of the labour room comprised three zones (Figure 8). By erecting the screen, we created a fourth (Figures 4,8). In the original zones, behaviours, hierarchies, and power dynamics were well-established among caregivers accustomed to working in this room. The new zone, however, had no fixed decorum. It emerged in response to the specific human and spatial elements of the situation.

In terms of required gatekeeping, there was a significant difference in privacy and protectedness between the bathroom and the screened birthspace. The bathroom has a heavy lockable door provides aural and visual protection and also contained a shower, and therapeutic showering is effective for pain management (69, 70). By contrast, the screen was light and easily moved, for it to function as a threshold to entry it required gatekeeping and collaborative agreement to respect its presence. Nonetheless, staff were very respectful and knocked by saying ‘knock knock’, always asking for permission to walk in. A factor in whether a curtain acts effectively as a boundary in a depth structure is its associated gatekeeping. The fabric felt like it offered me safety, but really, protection was created by the collaboration of the people in the room, especially Phillippa, who acknowledged and respected the boundary, making it real. Gatekeeping enforces thresholds which are demarcated by physical objects, within a depth structure.

Unlike the sanctum of the bathroom, the cloth was designed and implemented by me, and although I had very little control over lighting, temperature, or sound, I had perceived control over who was permitted to enter my new zone. The bathroom was already part of the physical structure of the hospital and labour ward, the rules of decorum are set within the institution and society at large: people do not generally enter a bathroom while someone else is using it, so privacy is virtually guaranteed. By contrast, the decorum of the screened birthspace was created by the collaborative behaviour of everyone in the room, led by Philippa, Becky, and V. On only one occasion did it feel like the ‘rules of play’ behind the screen were disrupted: when, prior to Becky's arrival, a clinician popped his head around and offered me pain relief. He offered sympathy (which in my experience accorded with disintegrative power) while later Becky offered only encouragement (which accorded with integrative power), she met groans with assertive yet supportive responses and framed the pain as a meaningful and expected part of the process. The screened space felt private and protected, my autonomy was respected, my body and its capacity for safe physiological birth were trusted, and the risks associated with the birth outside guidance were held by human beings, who used simple low-tech tools: hands, eyes, ears, and a handheld Doppler. Only at the very end of labour did a technology of surveillance enter the screened space, in the form of the ultrasound to check the position of twin 2, and even then, the ultrasound operators adhered to the social decorum of the screened birthspace and ‘knocked’ to enter.

### 5.4 Nested spaces

Both the bathroom and the screened birthspace inside the labour room were embedded or nested spaces: an enclosure within an already relatively private place. For me, this self-made extra layer of privacy was an important spatial configuration, yet such nested spaces are mentioned only rarely in the literature. The birthing pool, in particular, appears in studies as an architecture that offers a clear boundary. Shielded from interference and free to labour on their own terms (62, 71), ‘women who laboured in hospitals reported that the birth pools mitigated against harsh clinical environments and intervention by providing a space or territory that they could make their own’ (72). Similarly, Joyce's study participants identified ‘zones’ in hospital birth rooms which felt like they belonged to the staff, or their companions, or were their own jurisdiction (73). Joyce's participants also felt ‘the majority of the room was identified as belonging to the midwife … these women would have preferred a physical demarcation between the zones they perceived’ (73, p. 236). When birthing at home, people often retreat to the upper floors to seek solitude and distance from their companions and may restrict the access of midwives to specific rooms (73). According to Space Syntax analysis, the screened birthspace had a generous ‘step depth' and low visibility (54, 57, 58). The presence of the screen marked the boundary of a newly installed sanctum birth territory, contained within the relatively private labour room, which had both a door and a curtain, meaning there were three thresholds for intruders to cross between me and the public place of the corridor, all of which had gatekeepers who were not me.

### 5.5 Orientation away from the bed

The presence of the cloth oriented attention away from the bed, yet all the accoutrements of a 21st-century hospital remained immediately available. Prior to the erection of the screen, the hospital birth room conveyed risk (44), rather than empowerment (15). As Colin pointed out, when on the bed ‘in the view of the room’, we were the least expert people in our own birth experience. The layout of the 20th-century hospital birth room assumes the role of a patient for a labouring person, keeping her supine on the hospital bed (74, 75) while the prevailing biomedical model, often centred around the hospital bed, can hinder access to conditions for effective physiological birth. There has been a restrictive interpretation of woman-centred care as solely for low-risk people seeking a conventional birth (76). It can be perceived as an ‘exclusionary model’ that dismisses the needs of birthing people like myself, navigating complex social or medical circumstances. Sometimes, woman-centred care is also seen as carrying connotations of opposition to hospitals (77). Seeing a bed in the room can limit mobility (78). Yet here when new people entered, the screen drew attention. It sent a message that I had made decisions about this birth, and to Philippa, it signalled I wanted a particular type of birth: this person is clear about what she wants and will go to unusual lengths to get it.

In contrast to the ‘integrative power’ (22) upheld in the screened birthspace, the bed was a surveillance zone of ‘disintegrative power’ (22). Neurophysiological research shows that uncomfortable or disempowering birth spaces can trigger stress responses, disrupting labour (79, 80). The ‘Fear Cascade’ theory explains how the sympathetic nervous system responds to acute stress during labour (6, 62, 81), triggering the ‘Fight, Flight, or Freeze’ response through catecholamine release (62, 82). Adrenaline slows or stops labour and redirects blood away from the uterus, potentially leading to foetal distress (62). In a study by Mondy et al. (15), researchers observed passivity in many participants when they were in conventional labour rooms and suggested it may reflect a ‘freeze’ response. Experiences in the early part of labour: waiting to find out if the institution agreed I was in labour, being cannulated (which hurts) and frightened and annoyed by ineffective CTG could have resulted in less effective contractions. The passive response to threat also explains why I agreed to a caesarean section that I did not want.

### 5.6 The intersections between depth structure, people, and the institution

Although Philippa, V, and Becky's protective behaviour around the boundaries of the screened birthspace was a strong force in shaping my safe, satisfying birth, it may also be that the new layout of the room and the qualities of the cloth itself were an environment in which such behaviour was more likely. The birth environment can activate either the fight-or-flight response or the calm and connection system for both birthing people and caregivers (8, 30) argue caregivers such as doulas and midwives carry a high load of emotional labour (82) related to ideology, organisational culture, and interpersonal relationships (83), and part of this labour involves managing feelings generated by the environment. The hormone oxytocin supports successful physiological birth and increases trust, reduces fear and anxiety, as well as heightens caregiving qualities such as trust, generosity, openness, and empathy (30). Calm, warm spaces support oxytocin release, while threatening ones trigger stress (6, 86). The screened birthspace was warm-coloured and protective as Becky noticed, and the feelings of alignment with the environment could perhaps extend to my caregivers as well as myself. The depth structure and sensory qualities may have worked together to support a birth space that activated calm and connection for everyone present.

The individualised curation of the room when we erected the screen is rare but not unheard of. In the study of Mondy et al., four of the five people labouring in conventional hospital birth sessions quickly assumed the role of ‘patient’, they avoided taking up space, kept belongings neatly in corners, and accepted instructions without question (15). Joyce calls this model of spatial practice in birthing people ‘wait and transfer’ and notes that birthing people who occupy institutional spaces without curating them often do not move instinctively when labouring but may, for example, sit on the bed, waiting for the next thing to happen to them (41). This reflects literature on the complexities of negotiating the ‘patient’ role during labour (13, 84, 85). I purposefully did not wait, even when put into a triage room, where I used the curtains and bed as props to facilitate instinctive movements of labour. One person in the study of Mondy et al. did what I did: ‘Florence was the exception to the passive patient role. Despite giving birth in a highly medicalised environment, she redefined the space by bringing in family, personal belongings, and rearranging furniture to support an active, upright birth. Through these actions, she transformed the atmosphere, creating a sense of safety and satisfaction in her birth experience’ (15, p. 42).

Once we arrived in the labour room, my familiarity with the room from a previous admission meant the room had already become like home, I knew where everything was, and I had already conceptualised the various available zones of occupation and considered a spatial intervention to create a nested depth structure. Nonetheless, in my case, it took a lot of privilege, social capital, and knowledge, and even with these attributes, it almost did not ‘work’ to achieve my goals; it needed additional gatekeeping from dedicated caregivers to do its job. In Joyce's architectural study of spatial practices in birthing people, Urbinia was the only person to birth in a hospital who adapted the room. Her room was located in the most private part of the labour ward, and like me, Urbinia had familiarised herself with the room she requested in labour on an antenatal tour. The room Urbinia occupied also consisted of two spaces: a birthing space and an ensuite, ‘between which she moved freely’, moving furniture about as she required it (41, p. 550). Joyce calls this behaviour ‘curate and prosume’, and it is common in midwifery-led units and homebirth. Nothing is mentioned either by Joyce or Mondy et al. about the privilege, or social capital of Florence or Urbinia, although Florence had a detailed birth plan and a lot of family who ‘appeared to “fill” all the available spaces’ (15:42), which suggests she was knowledgeable and perhaps the family acted as gatekeepers. What captures the attention of birthing people and shapes their desires regarding birth spaces often lies within their existing knowledge (87, 88). And my knowledge was more architectural and spatial than most, thanks to a PhD and subsequent architectural and urban research in which I have explored and tested the phenomenon of depth structure.

## 6 Conclusion

This research explored, through a case study of a single high-risk birth, how spatial configurations in birth environments can shape experience and behaviour using a narrative walk-through and spatial analysis to uncover the relationships between individual and institutional power, arguing that space and care interact in complex ways to shape birth experiences. While one might not initially perceive the high-risk labour room where I delivered Julian as inconducive to ideal physiological birth conditions, it proved otherwise when supported by a simple, cost-effective architectural intervention and a respectful team. Based on my own previous theorising of depth structure, immediately upon entering the birth room, I curated it: found affordances and erected the cloth. As I laboured, I sought spaces that took me into progressively more internal and less public zones within this new depth structure. But without Becky's gatekeeping and Philippa's agreement, the screened birthspace lacked the privacy and protection I needed. This is evident in my agreement to return to the bed for extended monitoring, an experience of surveillance that led to the decision for a C-section, fuelled by the fear cascade. Later, Colin and I hid in the bathroom, and when Becky arrived, she turned off the light, halting the fear cascade and returning me to a path of physiological labour. With Becky's presence and Philippa's support for a birth outside guidance, I returned to the space behind the cloth, now more strongly defended, and had a safe and satisfying birth of my baby, Julian.

In creating a screened birthspace, the cloth did several things: it added step depth and reduced integration; blocked my view to the room, particularly the hospital bed and rescusitaires; reoriented the attention of people entering the room away from the bed; relocated me as the locus of birth into a territory which I had created and felt as though it ‘belonged’ to me; created a new place with decorum that has to be negotiated *ad hoc*; and sent a message that I was an active participant in shaping the space, exercising spatial agency rather than adhering to the norms of the room.

The context was favourable. The labour room itself already provided many of the birth environment conditions associated with safe, satisfying birth: it was private and acoustically separated, it had an en suite, most of the technological equipment except the resuscitaires, which arrived later, was concealed in domestic-style cupboards. Yet, the cloth created another zone in the depth structure of the room, an additional threshold in the gradation of publicness from the most private to the most public zones, which could be defended by my gatekeepers. I suggest it could be that the cloth itself, and the new zone created with emergent sociospatial decorum, was a tool which supported the caregivers in behaving as they did.

It is important to note that my capacity to design, implement, and maintain the cloth as a boundary in the depth structure of the birth room is a feature of my various forms of privilege. I could afford to hire an expert doula to advocate for me and my chosen spatial practices; I had detailed knowledge of both physiology of birth and of architecture; I was able to build mutually respectful relationships with staff who were (mostly) not tempted to behave in paternalistic ways.

### 6.1 Implications for practice and further research

The narrative presented here mirrors the spatial practices of birthing people before: moving between different spaces before settling in a final, more secluded area. In a hospital environment, such self-management can be facilitated through the availability of private and protected ‘suite rooms’ that offer multiple spaces. However, the implications of this research are that movable screens may also have a place in high-risk labour rooms, so birthing people can create additional layers of privacy in the spaces they occupy. However, power over such spatial interventions must be in the hands of those giving birth, and the new zones must be appropriately gatekept by caregivers. Perhaps who emplaces such screens (belong to the birthing person or the institution) plays an important contributing role in their impact.

These topics would bear further scrutiny, alongside research which fills the gaps identified in this paper's introduction: frequent lack of clear distinction between sociospatial structuring and aesthetics and limited empirical data on how the environment affects birth outcomes, experience, spatial practices, and clinician's practice in high-risk birth. The narrative insights from this paper also point towards the future development of a participatory method for analysing and adapting birth spaces, integrating lived experiences with theoretical spatial evaluation. Such a method could involve experts, staff, caregivers, and brithing people in analysing and shaping environments that support diverse forms of care. Overall, there is a pressing need for interdisciplinary, user-centred research that values all voices and reveals mechanisms around how space supports or hinders safe, satisfying birth.

## Data Availability

The datasets presented in this article are not readily available because the data used were in-depth interviews containing identifiable data, plus personal refelections, and may be made available upon request with participants' permission. Requests to access the datasets should be directed to j.clossick@londonmet.ac.uk.

## References

[1] Australian College of Midwives. Midwifery philosophy. (2020). Available online at: https://www.midwives.org.au/midwifery-philosophy-values (Accessed July 30, 2023).

[2] Buckley S. Ecstatic birth: nature’s hormonal blueprint for labor. Mothering Magazine. (2002) (111). Available from: Available online at: https://www.thevillagemidwife.com/wp-content/uploads/2017/04/Ecstatic-birth.pdf (Accessed March 01, 2025).

[3] Skrundz M Bolten M Nast I Hellhammer D Meinlschmidt G. Plasma oxytocin concentration during pregnancy is associated with development of postpartum depression. Neuropsychopharmacology. (2011) 36:1886–93. 10.1038/npp.2011.74 21562482 PMC3154107

[4] McKinnon K. The geopolitics of birth. Area. (2016) 48(3):285–91. 10.1111/area.12131

[5] Nilsson C Wijk H Höglund L Sjöblom H Hessman E Berg M. Effects of birthing room design on maternal and neonate outcomes: a systematic review. Health Environ Res Des J. (2020) 13(3):198–214. 10.1177/1937586720903689 PMC7364772 32077759

[6] Foureur M. Creating birth space to enable undisturbed birth. In: Fahy K Foureur M Hastie C, editors. Birth Territory and Midwifery Guardianship: Theory for Practice, Education and Research. Oxford, United Kingdom: Elsevier (2008). p. 57–77.

[7] Lefebvre H Enders MJ. Reflections on the politics of space. Antipode. (1976) 8(2):30–7. 10.1111/j.1467-8330.1976.tb00636.x

[8] Hammond A Foureur M Homer CSE. The hardware and software implications of hospital birth room design: a midwifery perspective. Midwifery. (2013) 30:825–30. 10.1016/j.midw.2013.07.013 23932739

[9] Foureur MJ Leap N Davis D Forbes I Homer CSE. Developing the birth unit design spatial evaluation tool (BUDSET): a qualitative study. HERD. (2010) 3(4):43–57. 10.1177/193758671000300405 21165851

[10] Stark MA Remynse M Zwelling E. Importance of the birth environment to support physiologic birth. J Obstet Gynecol Neonatal Nurs. (2016) 45(2):285–94. 10.1016/j.jogn.2015.12.008 26820356

[11] Behruzi R Hatem M Goulet L Fraser W Leduc N Misago C. Humanized birth in high risk pregnancy: barriers and facilitating factors. Med Health Care Philos. (2010) 13(1):49–58. 10.1007/s11019-009-9220-0 19669934

[12] Lepori B. Freedom of movement in birth places. Child Environ. (1994) 11(2):81–7. Available online at: https://www.jstor.org/stable/41514917

[13] Newburn M Singh D. Creating a Better Birth Environment: Women’s Views About the Design and Facilities in Maternity Units: A National Survey. London: National Childbirth Trust (2003).

[14] Hodnett ED Downe S Walsh D. Alternative versus conventional institutional settings for birth. Cochrane Database Syst Rev. (2012) (8):CD000012. 10.1002/14651858.CD000012.pub4 22895914 PMC7061256

[15] Mondy T Fenwick J Leap N Foureur M. How domesticity dictates behaviour in the birth space: lessons for designing birth environments in institutions wanting to promote a positive experience of birth. Midwifery. (2016) 43:37–47. 10.1016/j.midw.2016.10.009 27842228

[16] Lawrence A Lewis L Hofmeyr GJ Styles C. Maternal positions and mobility during first stage labour. Cochrane Database Syst Rev. (2013) (8):CD003934. 10.1002/14651858.CD003934.pub4 23959763

[17] Hammond AD Homer CE Foureur MJ. Friendliness, functionality and freedom: design characteristics that support midwifery practice in the hospital setting. Midwifery. (2017) 50:133–8. 10.1016/j.midw.2017.03.025 28432967

[18] Foureur MJ Leap N Davis DL Forbes IF Homer CE. Testing the birth unit design spatial evaluation tool (BUDSET) in Australia: a pilot study. HERD. (2011) 4(2):36–60. 10.1177/193758671100400205 21465434

[19] Hauck Y Rivers C Doherty K. Women’s experiences of using a snoezelen room during labour in Western Australia. Midwifery. (2008) 24(4):460–70. 10.1016/j.midw.2007.03.007 17659817

[20] Igarashi T Wakita M Miyazaki K Nakayama T. Birth environment facilitation by midwives assisting in non-hospital births: a qualitative interview study. Midwifery. (2014) 30(7):877–84. 10.1016/j.midw.2014.02.004 24656329

[21] Carolan-Olah M Kruger G Garvey-Graham A. Midwives’ experiences of the factors that facilitate normal birth among low risk women at a public hospital in Australia. Midwifery. (2015) 31(1):112–21. 10.1016/j.midw.2014.07.003 25132098

[22] Fahy KM Parratt JA. Birth territory: a theory for midwifery practice. Women Birth. (2006) 19(2):45–50. 10.1016/j.wombi.2006.05.001 16890902

[23] Sands G Evans K Spiby H Eldridge J Pallotti P Evans C. Birth environments for women with complex pregnancies: a mixed-methods systematic review. Women Birth. (2023) 36:39–46. 10.1016/j.wombi.2022.04.008 35431173

[24] Goldkuhl L Dellenborg L Berg M Wijk H Nilsson C. The influence and meaning of the birth environment for nulliparous women at a hospital-based labour ward in Sweden: an ethnographic study. Women Birth. (2022) 35(4):e337–47. 10.1016/j.wombi.2021.07.005 34321183

[25] Setola N Naldi E Cardinali P Migliorini L. A broad study to develop maternity units design knowledge combining spatial analysis and mothers’ and midwives’ perception of the birth environment. Health Environ Res Des J. (2022) 15(4):204–32. 10.1177/19375867221098987 PMC9520132 36165447

[26] Rados M Kovács E Mészáros J. Intimacy and privacy during childbirth: a pilot study testing a new self-developed questionnaire, the childbirth intimacy and privacy scale (CIPS). New Med. (2015) 1:16–24. 10.5604/14270994.1155328

[27] Nielsen JH Overgaard C. Healing architecture and Snoezelen in delivery room design: a qualitative study of women’s birth experiences and patient-centeredness of care. BMC Pregnancy Childbirth. (2020) 20:283. 10.1186/s12884-020-02983-z 32393297 PMC7216688

[28] Hodnett ED Downe S Walsh D. Alternative versus conventional institutional settings for birth. Cochrane Database Syst Rev. (2012) 2012(8):CD000012. 10.1002/14651858.CD000012.pub4 22895914 PMC7061256

[29] Sandall J Soltani H Gates S Shennan A Devane D. Midwife-led continuity models versus other models of care for childbearing women. Cochrane Database Syst Rev. (2016) (4):CD004667. 10.1002/14651858.CD004667.pub4 27121907 PMC8663203

[30] Hammond A Homer CSE Foureur M. Messages from space: an exploration of the relationship between hospital birth environments and midwifery practice. HERD. (2014) 7(4):81–95. 10.1177/193758671400700407 25303428

[31] Lothian JA. How do women who plan home birth prepare for childbirth? J Perinat Educ. (2010) 19(3):62–7. 10.1624/105812410X514459 21629387 PMC2920656

[32] Levy-Shiff R Lerman M Har-Even D. Maternal adjustment and infant outcome in medically defined high-risk pregnancy. Dev Psychol. (2002) 38(1):93–103. 10.1037/0012-1649.38.1.93 11806705

[33] Lindsay P. Creating normality in a high risk pregnancy. Pract Midwife. (2006) 9(1):17–20. 16425679

[34] Gupton A Heaman M Ashcroft T. Complicated and uncomplicated pregnancies: women’s perception of risk. J Obstet Gynecol Neonatal Nurs. (2001) 30(2):192–201. 10.1111/j.1552-6909.2001.tb01535.x 11308109

[35] Clauson MI. Uncertainty and stress in women hospitalized with high-risk pregnancy. Clin Nurs Res. (1996) 5(3):309–25. 10.1177/105477389600500306 8850774

[36] Walsh D Spiby H Dodwell M Dodwell M McCourt C Culley L Mapping midwifery and obstetric units in England. Midwifery. (2018) 56:9–16. 10.1016/j.midw.2017.09.009 29024869

[37] MacDorman MF Declercq E. Trends and state variations in out-of-hospital births in the United States, 2004–2017. Birth. (2019) 46(2):279–88. 10.1111/birt.12411 30537156 PMC6642827

[38] Homer CSE Cheah SL Rossiter C Dahlen HG Ellwood D Foureur MJ Maternal and perinatal outcomes by planned place of birth in Australia 2000–2012: a linked population data study. BMJ Open. (2019) 9(10):e029192. 10.1136/bmjopen-2019-029192 31662359 PMC6830673

[39] Danilack VA Nunes AP Phipps MG. Unexpected complications of low-risk pregnancies in the United States. Am J Obstet Gynecol. (2015) 212(6):809.e1–6. 10.1016/j.ajog.2015.03.038 26042957 PMC4728153

[40] Rashid M. The question of knowledge in evidence-based design for healthcare facilities: limitations and suggestions. Health Environ Res Des J. (2013) 6(4):101–26. 10.1177/193758671300600407 24089184

[41] Joyce S. Wait and transfer, curate and prosume: women’s social experiences of birth spaces architecture. Women Birth. (2021) 34(6):540–53. 10.1016/j.wombi.2020.11.003 33341363

[42] Lepori B Foureur MJ Hastie CR. Mindbodyspirit architecture: creating birth space. (2008).

[43] Bourgeault IL Sutherns R MacDonald M Luce J. Problematising public and private work spaces: midwives’ work in hospitals and in homes. Midwifery. (2012) 28(5):582–90. 10.1016/j.midw.2012.06.002 22925395

[44] Davis D Walker K. The corporeal, the social and space/place: exploring intersections from a midwifery perspective in New Zealand. Gend Place Cult. (2010) 17(3):377–91. 10.1080/09663691003737645

[45] Freeman L Adair V Timperley H West S. The influence of the birthplace and models of care on midwifery practice for the management of women in labour. Women Birth. (2006) 19(4):97–105. 10.1016/j.wombi.2006.10.001 17070742

[46] Setola N Naldi E Cocina GG Eide LB Iannuzzi L Daly D. The impact of the physical environment on intrapartum maternity care: identification of eight crucial building spaces. HERD. (2019) 13(1):137–52. 10.1177/1937586719826058 30767614

[47] Deleuze G Guattari F. A Thousand Plateaus: Capitalism and Schizophrenia. Massumi B, Translator. Minneapolis: University of Minnesota Press (1987).

[48] Merleau-Ponty M. Phenomenology of Perception. Smith C, Translator. London: Routledge Classics (2002). Original work published 1945.

[49] Ellis C Adams TE Bochner AP. Autoethnography: an overview. Forum Qual Sozialforsch Forum Qual Soc Res. (2011) 12(1):Art. 10.

[50] Richardson L. New writing practices in qualitative research. Sociol Sport J. (2000) 17(1):5–20. 10.1123/ssj.17.1.5

[51] Riessman CK. Narrative Methods for the Human Sciences. 1st ed. Thousand Oaks (CA): SAGE Publications, Inc (2008).

[52] Lefebvre H. The Production of Space. Nicholson-Smith D, Translator. Oxford (UK): Blackwell (1991).

[53] Soja EW. Thirdspace: Journeys to Los Angeles and Other Real-and-imagined places. Oxford (UK): Wiley-Blackwell (1996).

[54] Hillier B Hanson J. The Social Logic of Space. Cambridge: Cambridge University Press (1984). 10.1017/CBO9780511597237

[55] Clossick J. The depth structure of a London high street: a study in urban order (Doctoral thesis). London Metropolitan University, London (2017).Available online at: https://repository.londonmet.ac.uk/1278/

[56] Clossick J Colburn B. Design precepts for autonomy: a case study of kelvin Hall, Glasgow. In: Lewis P Holm L Santos SC, editors. Architecture and Collective Life. 1st ed. London: Routledge (2021). p. 11. eBook ISBN: 9781003118985.

[57] Turner A. Depthmap 4: A Researcher’s Handbook. London: Bartlett School of Graduate Studies, University College London (2004).

[58] Pachilova R Sailer K. Providing care quality by design: a new measure to assess hospital ward layouts. J Architect. (2020) 25(2):186–202. 10.1080/13602365.2020.1733802

[59] Haq S Luo Y. Space syntax in healthcare facilities research: a review. Health Environ Res Des J. (2012) 5(4):98–117. 10.1177/193758671200500409 23224810

[60] Altaweli R. Dynamics of Social, Cultural, and Spatial Dimensions on Childbirth Experiences in Three Jeddah Hospitals: A Mixed Methods Study [dissertation]. Cincinnati (OH): University of Cincinnati (2023).

[61] Naughton SL Harvey C Baldwin A. Providing woman-centred care in complex pregnancy situations. Midwifery. (2021) 102:103060. 10.1016/j.midw.2021.103060 34175656

[62] Stenglin M Foureur M. Designing out the fear cascade to increase the likelihood of normal birth. Midwifery. (2013) 29(8):819–25. 10.1016/j.midw.2013.04.005 23706980

[63] Corson P. Presentation given at the Spaces of Birth Workshop, London Metropolitan University. (2023). Available online at: https://urbandepth.research.londonmet.ac.uk/wp-content/uploads/2023/09/Transcript-Philippa-Corson.pdf (Accessed March 11, 2025)

[64] Dahan O. The riddle of the extreme ends of the birth experience: birthing consciousness and its fragility. Curr Psychol. (2023) 42:262–72. 10.1007/s12144-021-01439-7

[65] Hallsdorsdottir S Karlsdottir S. The primacy of the good midwife in midwifery services: an evolving theory of professionalism in midwifery. Scand J Caring Sci. (2011) 25:806–17. 10.1111/j.1471-6712.2011.00886.x 21480938

[66] Byrom S Downe S. She sort of shines’: midwives’ accounts of ‘good’ midwifery and ‘good’ leadership. Midwifery. (2010) 26(1):126–37. 10.1016/j.midw.2008.01.011 18375025

[67] Maclellan J. The art of midwifery practice: a discourse analysis. Midwifery Digest. (2011) 21:25–31.

[68] Lundgren I Karlsdóttir S Bondas T. Long-term memories and experiences of childbirth in a Nordic context: a secondary analysis. Int J Qual Stud Health Well-being. (2009) 4(2):115–28. 10.3402/qhw.v4i2.5008

[69] Declercq ER Sakala C Corry MP Applebaum S. Listening to Mothers II: Report of the Second National U.S. Survey of Women’s Childbearing Experiences. New York: Childbirth Connection (2006.]).

[70] Stark MA. Therapeutic showering in labor. Clin Nurs Res. (2013) 22(3):359–75. 10.1177/1054773812471972 23324894

[71] Maude RM Foureur MJ. It’s beyond water: stories of women’s experience of using water for labour and birth. Women Birth. (2007) 20(1):17–24. 10.1016/j.wombi.2006.10.005 17174165

[72] Feeley C Cooper M Burns E. A systematic meta-thematic synthesis to examine the views and experiences of women following water immersion during labour and waterbirth. J Adv Nurs. (2021) 77(7):2942–56. 10.1111/jan.14720 33464640

[73] Joyce S. Towards a new Architectural Understanding of Birth Spaces Grounded in Women’s Experiences of Giving Birth [dissertation]. Sheffield (UK): The University of Sheffield (2018).

[74] Walsh D. Part five: why we should reject the bed birth myth. Br J Midwifery. (2000) 8(9):580–3. 10.12968/bjom.2000.8.9.8075

[75] Janssen PA Klein MC Harris SJ Soolsma J Seymour LC. Single room maternity care and client satisfaction. Birth. (2000) 27(4):235–43. 10.1046/j.1523-536x.2000.00235.x 11251508

[76] Carolan M Hodnett E. With woman’ philosophy: examining the evidence, answering the questions. Nurs Inq. (2007) 14:140–52. 10.1111/j.1440-1800.2007.00360.x 17518826

[77] Van Teijlingen E. A critical analysis of the medical model as used in the study of pregnancy and childbirth. Sociol Res Online. (2005) 10(2):63–77. 10.5153/sro.1034

[78] Gould D. Subliminal medicalisation. Br J Midwifery. (2002) 10(7):418. 10.12968/bjom.2002.10.7.10583

[79] Ulrich RS Berry LL Quan X Parish JT. A conceptual framework for the domain of evidence-based design. Health Environ Res Des J. (2010) 4:95–114. 10.1177/193758671000400107 21162431

[80] Buckley SJ. Hormonal Physiology of Childbearing: Evidence and Implications for Women, Babies, and Maternity Care. Washington, D.C.: Childbirth Connection (2015). Available online at: https://nationalpartnership.org/wp-content/uploads/2023/02/hormonal-physiology-of-childbearing.pdf (Accessed April 10, 2025). 10.1891/1058-1243.24.3.145 PMC4720867 26834435

[81] Lederman E Lederman RP Work BA Jr McCann DS. Maternal psychological and physiologic correlates of fetal-newborn health status. Am J Obstet Gynecol. (1981) 139(8):956–8. 10.1016/0002-9378(81)90447-3 7223798

[82] Hunter B. Emotion work in midwifery: a review of current knowledge. J Adv Nurs. (2001) 34(4):436–44. 10.1046/j.1365-2648.2001.01772.x 11380710

[83] Hunter B. Conflicting ideologies as a source of emotion work in midwifery. Midwifery. (2004) 20(3):261–72. 10.1016/j.midw.2003.12.004 15337282

[84] Walsh D. Improving Maternity Service. Small is Beautiful: Lessons for Maternity Services from a Birth Centre. Oxford: Radcliffe Publishing (2006).

[85] Hunt S Symonds A. The Social Meaning of Midwifery. Basingstoke: MacMillan (1995).

[86] Uvnäs-Moberg K Arn I Magnusson D. The psychobiology of emotion: the role of the oxytocinergic system. Int J Behav Med. (2005) 12:59–65. 10.1207/s15327558ijbm1202_3 15901214

[87] Singh D Newburn M. Feathering the nest: what women want from the birth environment. RCM Midwives. (2006) 9(7):266–9. 16886787

[88] van Teijlingen ER Hundley V Rennie AM Graham W Fitzmaurice A. Maternity satisfaction studies and their limitations: “What is, must still be best”. Birth. (2003) 30(2):75–82. 10.1046/j.1523-536x.2003.00224.x 12752163

